# Regret and Other Emotions Related to Decision-Making: Antecedents, Appraisals, and Phenomenological Aspects

**DOI:** 10.3389/fpsyg.2021.783248

**Published:** 2021-12-16

**Authors:** Olimpia Matarazzo, Lucia Abbamonte, Claudia Greco, Barbara Pizzini, Giovanna Nigro

**Affiliations:** Department of Psychology, University of Campania “Luigi Vanvitelli”, Caserta, Italy

**Keywords:** regret, decision-making, post-decision emotions, antecedents, appraisals, phenomenology

## Abstract

**Objectives:** The mainstream position on regret in psychological literature is that its necessary conditions are agency and responsibility, that is, to choose freely but badly. Without free choice, other emotions, such as disappointment, are deemed to be elicited when the outcome is worse than expected. In two experiments, we tested the opposite hypothesis that being forced by external circumstances to choose an option inconsistent with one’s own intentions is an important source of regret and a core component of its phenomenology, regardless of the positivity/negativity of the post-decision outcome. Along with regret, four post-decision emotions – anger toward oneself, disappointment, anger toward circumstances, and satisfaction – were investigated to examine their analogies and differences to regret with regard to antecedents, appraisals, and phenomenological aspects.

**Methods:** Through the scenario methodology, we manipulated three variables: choice (free/forced), outcome (positive/negative), and time (short/long time after decision-making). Moreover, we investigated whether responsibility, decision justifiability, and some phenomenological aspects (self-attribution, other attribution, and contentment) mediated the effect exerted by choice, singularly or in interaction with outcome and time, on the five emotions. Each study was conducted with 336 participants, aged 18–60.

**Results:** The results of both studies were similar and supported our hypothesis. In particular, regret elicited by forced choice was always high, regardless of the valence of outcome, whereas free choice elicited regret was high only with a negative outcome. Moreover, regret was unaffected by responsibility and decision justifiability, whereas it was affected by the three phenomenological dimensions.

**Conclusion:** Our results suggest that **(1)** the prevailing theory of regret is too binding, since it posits as necessary some requirements which are not; **(2)** the antecedents and phenomenology of regret are broader than it is generally believed; **(3)** decision-making produces a complex emotional constellation, where the different emotions, singularly and/or in combination, constitute the affective responses to the different aspects of decision-making.

## Introduction

The well-established psychological concept of regret based on decision theories strongly links the genesis of this emotion to the factual or counterfactual comparison between the actual outcome resulting from the option chosen in decision-making and the better outcome deriving from the foregone option (Bell, 1982; Loomes and Sugden, 1982; Tsiros, 1998; Roese and Summerville, 2005; van Dijk and Zeelenberg, 2005; Zeelenberg and Pieters, 2007; Beike et al., 2009). The corollary of this conception is the assumption that decision-makers choose freely and, thus, they have control over their actions and responsibility for their choices. Self-blaming for a bad outcome deriving from a wrong decision and the desire to undo this decision are considered as specific characteristics of the phenomenology^1^ of regret and as a discriminant criterion for distinguishing regret from other decision-related emotions, such as disappointment (Inman et al., 1997; Zeelenberg et al., 1998a,b; Gilbert et al., 2004).

It is worth noting that research on regret has come to a standstill in the last years, maybe because the wide consensus on the theory described above has led to the idea that the knowledge of this emotion does not need to be further developed. However, in this paper, we aimed to point out that the prevailing theory has not considered that an important source of regret is represented by forced choices, that is, by choices due to the constraint of external circumstances (e.g., physical accidents, indigence, family pressures, and deaths) that compel people to choose an option inconsistent with their intentions. A corollary of this assumption is that forced choices can produce regret even when their outcome is positive. Moreover, we aimed to show that the phenomenology of the regret elicited by forced choices is characterized by the awareness of having made an unsatisfactory but unavoidable choice. Finally, we aimed to reconsider analogies and differences between regret and other post-decision emotions.

In the following sections, the state of the art of the literature on regret and other decision-related emotions is reviewed, with particular reference to the aspects that have been investigated in our studies.

### Theoretical Background

A widely accepted definition posits that regret “is a comparison-based emotion of self-blame, experienced when people realize or imagine that their present situation would have been better had they decided differently in the past” (Zeelenberg and Pieters, 2007, p. 7). This conception originates from the *regret theory* built by rational decision theorists in Economics (Bell, 1982; Loomes and Sugden, 1982). According to this theory, decision-makers evaluate the outcome of the chosen alternative by comparing it with the outcome of the nonchosen alternative(s): Regret and rejoicing are the terms used to define the result of the unfavorable and favorable comparison, respectively. People tend to avoid choices that could generate regret.

Psychological research has expanded the economic conception of regret by highlighting that the comparison can occur not only at the factual but also at the counterfactual level, by imagining the possible outcomes of the foregone options (Kahneman and Miller, 1986; Kahneman, 1995; Roese, 1999; van Dijk and Zeelenberg, 2005). Moreover, such a research has also investigated structural features of the regrettable decisions (e.g., deciding of acting vs. not acting; time effect on action-based regret and inaction-based regret, see below), appraisals (van Dijk and Zeelenberg, 2002), phenomenology (e.g., Zeelenberg et al., 1998a; Marcatto and Ferrante, 2008; Summerville and Buchanan, 2014; Buchanan et al., 2016), behavioral consequences (of both anticipated and experienced regret; e.g., Mellers, 2000; Tsiros and Mittal, 2000; Zeelenberg et al., 2001; Marcatto et al., 2015; Davidai and Gilovich, 2018), and modes of regulation of regret (e.g., Inman, 2007; Zeelenberg and Pieters, 2007; Seta et al., 2008).

For example, many studies have been devoted to establish whether actions are more intensely regretted than inactions (see for a review, Feldman et al., 2020). The debate stemming from the different positions has highlighted relevant phenomena, such as the temporal pattern of regret (Gilovich and Medvec, 1994, 1995; Gilovich et al., 1998), according to which actions are more regretted in the short term, whereas inactions are more regretted in the long term. Another phenomenon is the importance of the context in which decisions are made, which makes sometimes actions and sometimes inactions more regrettable (Seta et al., 2001; Zeelenberg et al., 2002; Beike et al., 2009; Morrison and Roese, 2011; Seta and Seta, 2013; Feldman, 2020).

The pivotal points of the conception of regret shared by the above-mentioned studies are personal agency and responsibility, which are considered as structural features and central appraisals of regret. Consequently, the major components of its phenomenology are held to be self-blame for a bad decision and the desire to undo this decision. Similarly, since regret is assumed to derive from controllable events, its behavioral function is thought to be to modify the negative outcomes of the decision, when possible, or the type of choice in the future. These features are considered as a discriminant criterion for distinguishing regret from other negative emotions linked to decision-making, such as disappointment.

Indeed, the *disappointment theory* formulated by decision theorists (Bell, 1985; Loomes and Sugden, 1986) postulates that disappointment originates from an outcome that is worse than expected and that would have been better in a different state of the world. Consequently, the point of reference of regret is the nonchosen option; the point of reference of disappointment is the expected but unrealized outcome within the same chosen option.

A consistent body of research has tried to substantiate this distinction psychologically by showing that regret and disappointment have different patterns of appraisal, counterfactual thoughts, phenomenology, and behavioral consequences (see for reviews, Zeelenberg et al., 1998a, 2000a; van Dijk and Zeelenberg, 2002; George and Dane, 2016).

Although psychological research acknowledges that other emotions, besides regret and disappointment, can originate from decision-making (e.g., anger, sadness, satisfaction, and happiness), regret is considered unique for its relation with choice and responsibility (e.g., Roese and Summerville, 2005; Zeelenberg and Pieters, 2007; Buchanan et al., 2016; Davidai and Gilovich, 2018).

Compared to the position that ties regret to free choice and responsibility, the position that does not consider these requirements as essential, which we adopted in our research, is definitely not dominant. In addition to philosophers, such as Rorty (1980), Solomon (1983), and Taylor (1985), according to which regret can also stem from events beyond personal control or from choices for which no alternatives are available, in psychology, this position has been advanced by Landman. She states that it “is appropriate to speak of regret both with reference to one’s own free and voluntary acts (or omissions) and also with reference to acts over which one had no personal control” (Landman, 1987, p. 151). Subsequently, she defines regret as a cognitive and emotional negative state, which can be generated by a wide class of antecedents ranging from misfortunes to mistakes and from voluntary actions and omissions to uncontrollable and accidental events (Landman, 1993, p. 27).

Connolly and colleagues have supported this position through some scenario-based experiments (Connolly et al., 1997; Ordóñez and Connolly, 2000) where they manipulated the agent of decision-making (self vs. computer) and the outcome (better than, worse than, similar as the *status quo*). On the whole, their findings show that responsibility can amplify regret but is not a necessary condition for its genesis and its experience, since regret can also be elicited by external decision-makers, such as a computer. Regret, as well as disappointment, increases especially when the outcome is worse than the *status quo*. Moreover, Connolly and Reb (2005) and Connolly and Butler (2006) criticized the economic conceptions of regret and disappointment as being inadequate to account for genuinely felt emotions resulting from decisions. They state that experienced emotions of regret and disappointment are similar to a greater extent than rational decision theorists assumed and that emotional reactions to choice outcomes are better understood in term of negative (regret, disappointment, and sadness) vs. positive (rejoicing, elation, and happiness) emotion clusters.

An attempt to conciliate the divergent positions about the role of agency and responsibility in the genesis and experience of regret has been the decision justification theory (DJT, Connolly and Zeelenberg, 2002). According to DJT, regret stems from two types of antecedents and related appraisals, which are not necessarily co-present: a comparatively bad outcome and a bad decision-making. Consequently, the theory posits that the feeling of self-blame, which seems to be unique to the regret phenomenology, compared to other decision-making-related emotions, is associated with the awareness of having decided badly (Pieters and Zeelenberg, 2005), that is, in a rash or unjustified way. On the contrary, regret decreases or vanishes in virtue of the awareness that the decision process has been careful and accurate though its outcome has been negative, as some studies have demonstrated (Inman and Zeelenberg, 2002; Kwong et al., 2013; Towers et al., 2016; Verbruggen and De Vos, 2020).

However, an experimental study of Matarazzo and Abbamonte (2008) failed to find that the justifiability of the choice diminished the intensity of regret. Instead, this study revealed that being forced by external circumstances to choose an option different from the one desired generates more intense regret than the one produced by a free choice.

Interestingly, some studies on real life or career regrets have questioned the relationship between the intensity of regret and the controllability of the eliciting events. For example, Wilkinson et al. (2015) found that while the level of regret for controllable events varied as a function of individual self-esteem, the one for uncontrollable events was always high. In the career domain, Wrzesniewski et al. (2006), Berg et al. (2010), and Newton et al. (2012) found more intense regrets among those who had not chosen their current occupation, due to social or family constraints, than among those who had chosen it.

In summary, although some studies have challenged the idea that free choice and responsibility are a necessary condition for the onset of regret, to the best of our knowledge, there are no experimental studies that have systematically investigated the hypothesis that forced choice is an important source of regret and a core component of its phenomenology, regardless of the valence of the post-decision outcome. The studies presented here address this issue. As we will specify in the following section, we use the oxymoron “forced choices” to designate the choices for which no alternatives are available. In these types of choices, the decision-maker is not an external agent, as in the studies conducted by Connolly and colleagues (Connolly et al., 1997; Ordóñez and Connolly, 2000), but the individual herself. Nevertheless, decision-making is not free because, due to external or internal impediments or constraints, she cannot or feels she cannot choose the desired option.

### Regret and Free vs. Forced Choice: The Rationale of the Studies

The idea behind the present studies is that decision-making does not imply as a necessary condition that individuals decide freely. Indeed, people can be forced by external circumstances to choose an option that is inconsistent with one’s own intentions. In our opinion, this would elicit regret *per se*, independently of the outcome and the justifiability of decision-making, since the forced choice implies giving up the initial and preferred option. This effect should be particularly robust in self-relevant domains, due to the subjective importance of the preferred but not-chosen option. The “chosen” option, as a substitute for the former, would be inherently less attractive even if a positive outcome were derived from it. Moreover, in accordance with the “Zeigarnik effect” (1927–1935), the option that had to be given up and was not carried out tends to remain in the mind because of the psychological state of tension it generated, which was not resolved by the behavior. As Savitsky et al. (1997) pointed out, the persistence of unaccomplished tasks is likely to elicit regret. Free choice, on the contrary, should elicit regret only when decision-making results in a negative outcome. Consequently, regret stemming from forced choice should be more intense than the one derived from free choice because in the latter case it is moderated by the outcome, whereas in the former the outcome is deemed irrelevant.

More specifically, the main hypothesis that was tested in our studies is the following: A forced choice should elicit the same high intensity of regret both in case of positive and negative outcome, whereas a free choice should produce more intense regret with a negative than a positive outcome.

A corollary of this hypothesis concerns the characteristics of the phenomenology of regret: In the case of free choice, it should be marked by self-blame and the sense of personal responsibility; whereas when the choice was forced, it should be characterized by the awareness of having made an unwanted but inevitable choice. Consequently, responsibility should be not considered as a necessary requisite for the onset and the subjective experience of forced choice-related regret. Moreover, even the justifiability of the decision, which should decrease the intensity of regret (Connolly and Zeelenberg, 2002), should not be relevant in the case of forced choice, because here regret should focus on the option one was forced to give up, not on the accuracy of decision process. In our studies, we tested specifically if these two appraisal dimensions (responsibility and decision justifiability) are necessary for the onset of regret.

Our hypothesis and their corollaries, which question the unicity of the genesis and experience of regret, raise the question of whether and on what criteria regret can be differentiated from other post-decision emotions.

Many authors support the hypothesis that emotions arising from decision-making have specific antecedents, patterns of appraisal, and phenomenology (see “Theoretical Background”). However, other authors (Connolly and Butler, 2006) have pointed out that it would be more appropriate to classify these emotions, at least when they are self-reported, into two clusters: one formed by positive emotions (rejoicing, elation, happiness) and the other by negative emotions (regret, disappointment, sadness, and self-blame). Our position is somewhere in between these two. We believe that it is generally arbitrary to establish strict boundaries between emotions and that experiencing multiple or mixed emotions is the norm rather than the exception (Sabini and Silver, 2005; Heavey et al., 2017; Watson and Stanton, 2017). However, we assume that there is, at least in principle, a partial or fuzzy correspondence between emotional lexicon and emotional experience (Sini et al., 2014; Scherer and Fontaine, 2019) and between everyday life emotions, such as those investigated in the present studies, and theoretical emotions (Clore et al., 1987).

We adopt the perspective of appraisal theories (Frijda, 1986; Lazarus, 1991; Roseman, 2011; Lerner et al., 2015), according to which emotions are complex and targeted affective responses, which are based on appraisal dimensions and include experiential content and action tendencies. Hence, once emotions are activated, they lead us to interpret environmental events in line with the eliciting appraisals and to behave in order to pursue emotion-based goals. Thus, investigating the phenomenology of emotions is crucial to understand whether and to what extent different emotional words designate different emotional experiences, especially in situations from which a wide range of emotions can derive and there are many available labels to describe them.

We assume that an important decision made in a self-relevant domain results in a broad emotional reaction, where the composing emotions capture different aspects linked to decision-making both in overlapping and in mutually independent ways, so that each of them should have a specific configuration.

Along with regret, we investigated four emotions: disappointment, anger toward oneself, anger toward circumstances, and satisfaction. We focused our analysis on antecedents, and some appraisal and phenomenological aspects. In addition to disappointment, that is, the widely studied emotion that is closest to regret, the other emotions were chosen on the basis of the following criteria. Satisfaction, the only positive emotion, has been selected because it should increase with positive outcome. Moreover, since satisfaction refers to the affective reaction to one’s well-done actions (Mellers, 2000), it does imply ego-involvement and thus should increase with free choice and decision justifiability. Anger has been included because it can be elicited by obstacles that prevent the achievement of a goal, by frustration, and by wrong or stupid actions (Izard, 1991); such aspects are involved in forced choice, negative outcome, and bad (free) choice, respectively. Two types of anger have been distinguished as a function of the internal or external direction of this emotion, depending on the nature of its antecedents, appraisals, and phenomenology. Anger toward oneself arises from the self-attribution of a poor result and involves self-blame for having taken the wrong decision. Accordingly, it implies subjective responsibility and was supposed to increase with free choice and bad outcome, while decision justifiability should decrease it. Anger toward circumstances stems from the perception of an obstacle that interferes with one’s goals: Thus, it should increase with forced choice and bad outcome and entail external attribution of one’s situation. Disappointment should augment with forced choice and bad outcome for which one does not feel responsible. The two latter emotions should be not affected by decision justifiability because they should be focused on outcome rather than on choice.

Based on this analysis, we predicted some overlap between disappointment, regret, and anger toward circumstances, whereas the unique features attributed to regret should rather characterize anger toward oneself. In our view, the specificity of the nature of regret is that it can be elicited by opposite antecedents which, in turn, should generate contrasting subjective experiences.

Since we set out to systematically test our hypothesis about the importance of forced choice on the most relevant phenomena highlighted by research on regret, we also included in our investigation two well-known phenomena: the effect of the temporal dimension and of the type of behavior (action or inaction) resulting from decision-making on the intensity of regret.

We examined the first issue by considering the time elapsed from decision-making (a few days vs. a year) as a moderator of the effect of the type of choice. Instead, to investigate the second issue, we conducted two similar studies with two different samples: The only difference between them was that in the first study, the result of decision-making was an inaction, whereas in the second was an action.

According to the temporal theory of regret (Gilovich and Medvec, 1994, 1995), in the study on inaction, regret should be more intense in the long than in the short term, whereas in the study on action, the opposite pattern should be found. However, we left open the hypothesis of whether this pattern also concerns the forced choice-related regret, and we did not formulate specific hypotheses on the other emotions taken into consideration.

In the present studies, we adopted the scenario method, a widely used tool in the experimental studies on regret (Connolly et al., 1997; Zeelenberg et al., 1998b,c; Ordóñez and Connolly, 2000, studies 2 and 3; Zeelenberg et al., 2000b 2002). This method has the advantage of allowing easy experimental manipulation without running into ethical issues arising from putting participants in relevant real-world situations likely to generate distress or other intense emotional states. As well as other methods based on perspective taking, scenarios rely on the assumption that participants put themselves in the shoes of the protagonist and attribute to her/him what they would likely feel or think in a similar situation.

In general, studies based on perspective taking (regardless of the object of investigation and the type of technique used: scenario, photo, videotape, etc.) have used two types of instructions: *imagine-self* instructions and *imagine-target* instructions (Davis et al., 2004). In the first case, the instructions asked participants to put themselves in the place of the protagonist and imagine what they would feel in that situation. In the second case, the instructions asked participants to imagine what the protagonist was thinking or feeling.

Although some studies have found that *imagine-self* instructions produced more emotional involvement than *imagine-target* instructions (Batson et al., 1997; Davis et al., 2004, study 1), other studies (Davis et al., 2004, study 2; Avenanti et al., 2006; Chambers and Davis, 2012) have found no difference between the two types of instructions. They rather suggest that perspective taking is the natural way to interact with others, unless it is inhibited by specific instructions.

In regret studies, both instructions were used, particularly the *imagine-target* instructions.

We used this type of instructions in our studies by asking participants to imagine as vividly as possible what the protagonist of the scenario was feeling and thinking.

### The Studies

To test our hypothesis and its corollaries, two studies were conducted, each of them based on the scenario method. In both studies, the scenario protagonist had to make an important decision for her career. In the first study, the decision results in an inaction: The protagonist does not change her job position. In the second, the decision results in an action: She decide to change job.

In all scenarios, the outcome of the foregone option was known to the scenario protagonist: Thus, a requirement of rational decision theories was fulfilled. Actually, the protagonist could make a factual comparison between the consequences of both the chosen option and the nonchosen option, but he could also make a counterfactual comparison between his current situation (sometime after the decision) and what it might have been if he had made (or could have made) a different choice.

**Figure 1:**
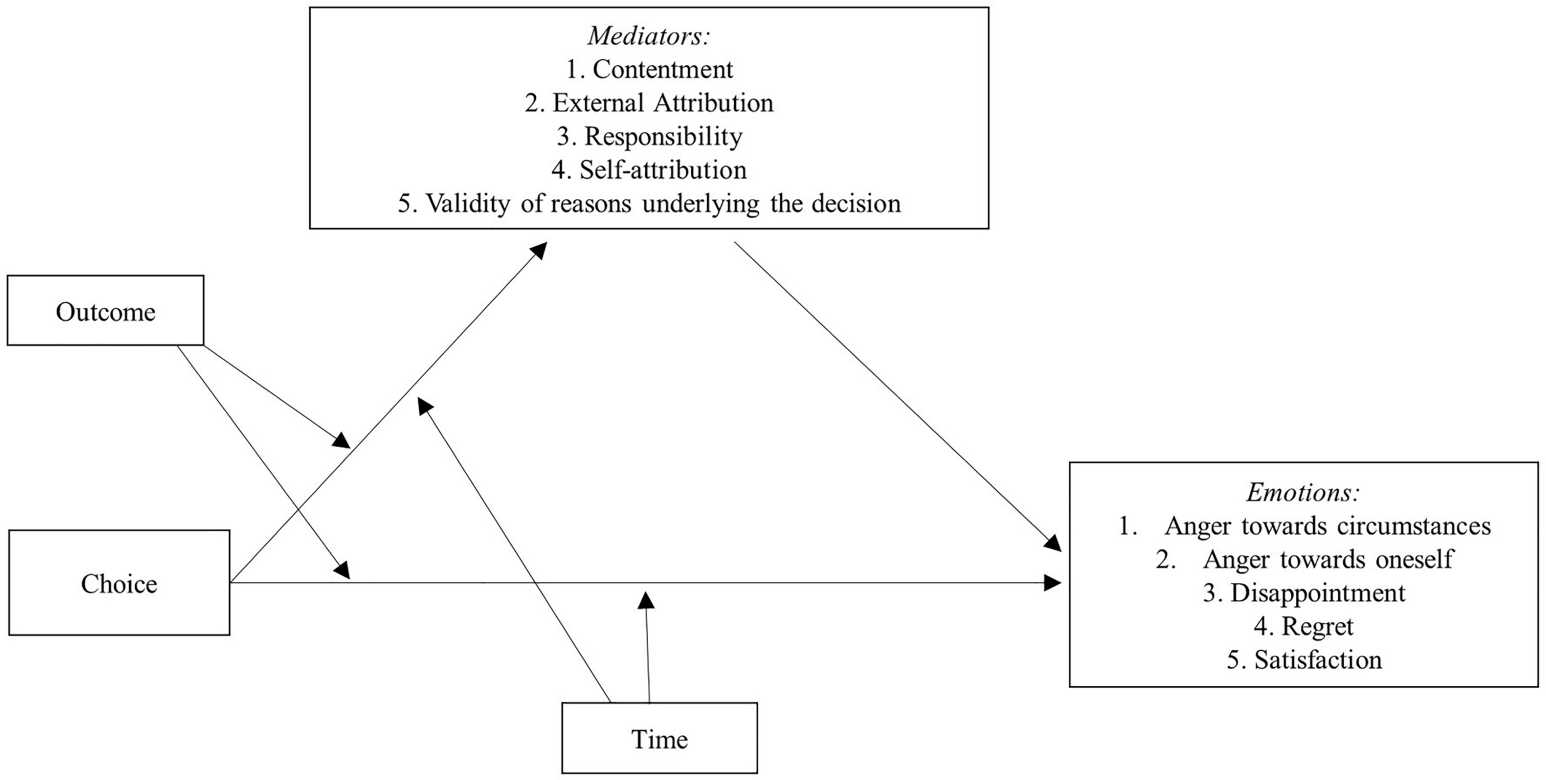
Model 10 of PROCESS macro.

For each study, we used five moderated mediation models (depicted in Figure 1), one for each of the five emotions taken into account, to test our hypothesis. In each model, choice (forced vs. free) was included as independent variable; outcome (negative vs. positive) and time (long term vs. short term after decision-making) were included as moderators; some intervening variables, that is, decision justifiability, responsibility, and phenomenological aspects (see below for their description), were included as mediators; each of the five emotions was included as dependent variable. According to the tested hypothesis, choice, singularly and/or by interacting with the moderators, should affect emotion through the mediation of the intervening variables. In other words, the effect of choice on emotion could vary in function of moderators and should be exerted through an indirect way, by means of the effects on the intervening variables.

These variables were assessed (see Materials and Procedure section) by means of one direct question for decision justifiability (i.e., to what extent the scenario’s protagonist judged the reasons underlying his own decision as valid) and for responsibility (i.e., to what extent she felt responsible for her choice), respectively. Instead, the phenomenological aspects were assessed by means of 14 questions, concerning feelings and factual and counterfactual thoughts about the type of choice made, its context, its degree of freedom, and its consequences. These questions were formulated for taking into account three dimensions: self-attribution, external attribution, and contentment. Note that we did not take into account all the aspects of the emotion phenomenology, but only those we judged relevant for investigating the subjective repercussions deriving from the type of choice. More specifically, we considered neither the intentions nor the action tendencies of the protagonist.

We expected that, for both studies, the independent variable (choice) and the moderators (outcome and time) should differently affect both the five emotions and the mediators and that, in turn, the latter should affect the five emotions differently. Thus, choice should exert conditional (in interaction with moderators) direct and indirect (through mediators) effects on the five emotions. In the previous section, we delineated some of the principal effects we predicted and the reasons underlying these predictions.

We conducted both studies in the career domain, which has been considered as one of the main domains where regret can be produced (Roese and Summerville, 2005; Sullivan et al., 2007; Verbruggen and De Vos, 2020). The scenarios presented two typical situations: deciding whether to accept a work assignment abroad for a certain period (study 1) and deciding which career to choose (study 2). They were conducted with employees of a wide age range (18–60 years): In this way, we tried to foster the identification of the participants with the protagonists of scenarios and to increase the ecological validity of the studies. In addition, the wide age range allowed us to investigate possible age-related differences in emotional reactions to the proposed decision situations.

Finally, it is worth specifying that in Italian, there are two terms corresponding to English regret, namely, *rimpianto* and *rammarico*, which are often used as synonyms, though their meaning varies slightly depending on the dictionaries (e.g., the Treccani Dictionary associates *rammarico* mainly with inaction, while the Garzanti Dictionary mainly associates *rimpianto* with inaction). We decided to select the most frequently occurring term on the web, which is *rimpianto*. For disappointment, the corresponding Italian term is *delusione*.

## Study 1

The study 1 investigated regret and other emotional reactions stemming from inaction.

### Method

#### Design and Participants

A 2 (choice: free vs. forced) × 2 (outcome: negative vs. positive) × 2 (time: short term vs. long term) between-subject design was carried out. Gender and age were considered as covariates. Three hundred thirty-six volunteer (unpaid) participants were recruited from different companies, sports centers, and universities in Campania. They were equally distributed by gender (168 males and 168 females), were aged between 18 and 60 (Mean = 33.45; S.D. = 10.82), and were randomly assigned to one of the 8 experimental conditions (*n* = 42 for each condition). All participants were employed. Among the students, only student workers were selected. Most of the participants (63.1%) had a high school diploma, 28.9% had a college degree, and 8% had completed compulsory education.

To determine the sample size, an *a priori* power analysis for ANCOVA on the five emotions taken into account was performed by using G*Power (Faul et al., 2007). To detect a medium-small effect size (*f* = 0.20) and achieve a power of (1-β) of 0.95 with an error probability of 0.05, a minimum sample size of 327 participants was required. We decided to increase the number of participants to 336 in case we had to exclude someone for incomplete responses. Actually, no missing data were found. All participants executed the experimental task individually, at the place where they were recruited. They gave their informed consent before starting the experiment.

#### Materials and Procedure

In conformity with the experimental conditions, eight scenarios were built with the same structure and three sources of variation: type of choice (free vs. forced), outcome (negative vs. positive), and time (consequences of decision-making evaluated in long vs. short term). In all scenarios, the protagonist is an executive of a company who has to decide whether to accept the offer to manage for 2 years a branch that the company is planning to open abroad. The job is very well paid, and the sector in which the branch will operate is very interesting; moreover, the protagonist is offered the possibility of a significant career advancement once (s)he returns. In all scenarios, decision-making results in inaction: The protagonist decides to stay in the same workplace, and the assignment is given to a colleague. In the free choice conditions, the protagonist does not have any constraints: (s)he is aware that the proposal to manage the branch abroad is a great opportunity for her career but prefers to remain in her workplace.

In the forced choice conditions, a physical accident prevents the protagonist from choosing the desired option (going to manage the company branch abroad) and (s)he is therefore forced to choose to stay in the same workplace. In the positive outcome conditions, the protagonist gets a career advancement and a small pay raise. In the negative outcome conditions, the company decides to downsize some areas of production, including the one where the protagonist works, because the costs of opening the foreign branch have implied a restructuring of activities. Consequently, any possibility of career advancement disappears and the protagonist’s position in the company becomes much more marginal. In both types of outcomes, the colleague who has gone abroad is very happy with his/her work. The outcome is evaluated a few days (short term condition) or 1 year (long term condition) after the choice. For each experimental condition, in half of the scenarios the protagonist is a woman; in the other half, it is a man. However, the scenarios were assigned randomly to the participants, without doing any matching between the gender of the protagonist and the one of the participant.

In all experimental conditions, participants received a two-page booklet, in the first of which one of the eight short stories was reported. After reading the scenario, they were asked to imagine as vividly as possible the thoughts and feelings of the protagonist, and then to evaluate, on a nine-point scale (1 = not at all; 9 = very much):

- how important she felt the object of the decision was for her life (“how important does the protagonist feel the decision whether to accept or refuse to manage a company branch abroad was for her life?);
- whether she perceived her decision as a free choice (“To what extent does the protagonist feel the choice of not accepting to manage a company branch abroad was a free choice?);
- whether she felt responsible for her own choice (“To what extent does the protagonist feel responsible for not accepting to manage a company branch abroad?”);
- whether she judged her decision as justifiable (“To what extent does the protagonist judge the reasons for not accepting to manage a company branch abroad as valid?”);
- whether she judged her present working condition as positive (“To what extent does the protagonist judge her present working condition as positive?”).

In this way, the effectiveness of the experimental manipulation was checked and two of the putative mediation variables between type of choice and regret, that is, responsibility and decision justifiability, were assessed. Henceforth, the five variables will be labelled as Intermediate Variables (InVs).

Successively, participants evaluated on a nine-point scale (1 = not at all; 9 = very much) how intensely the protagonist was supposed to feel five emotions: anger toward circumstances, anger toward oneself, disappointment, regret, and satisfaction.

**Figure 2:**
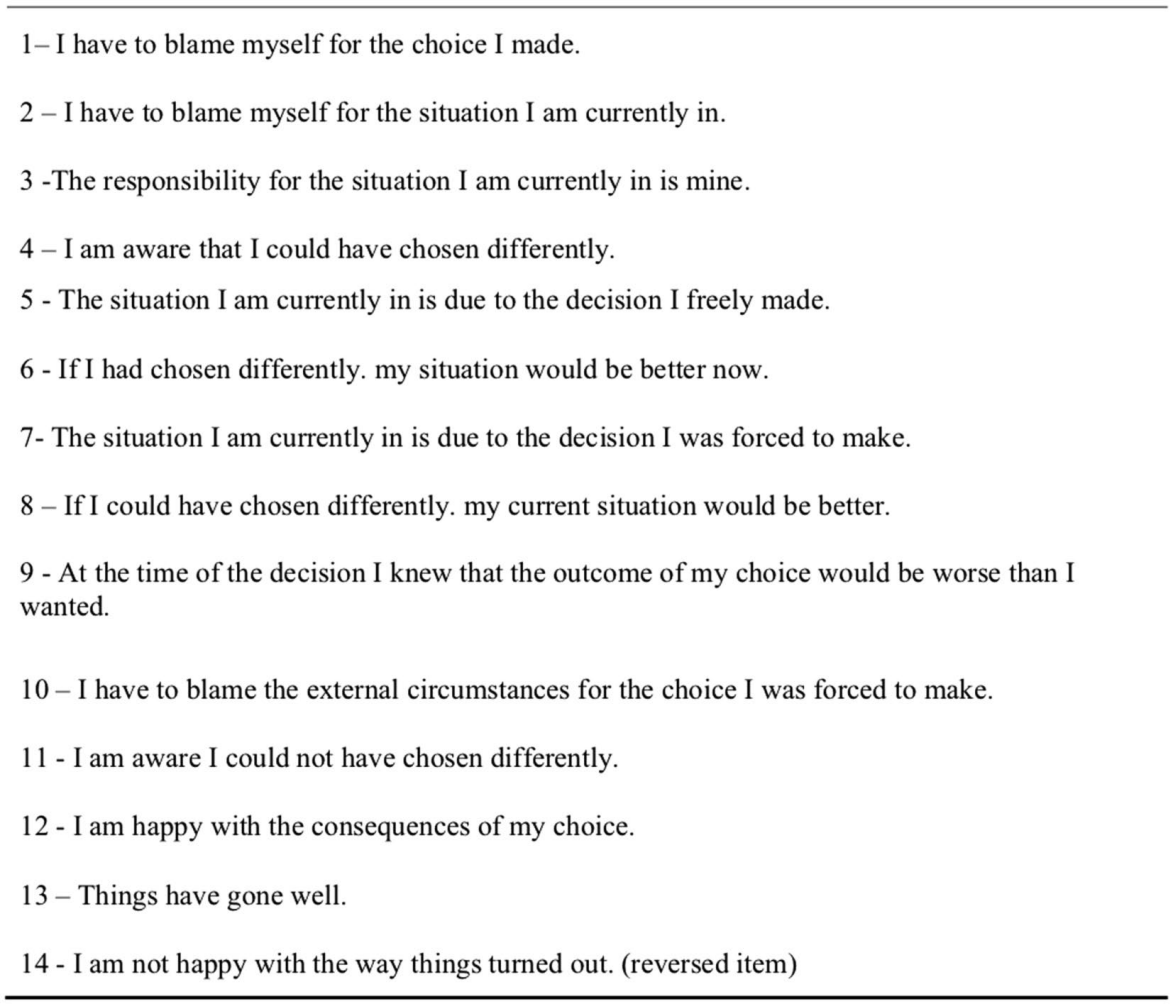
Items of the phenomenology questionnaire.

Finally, they estimated on a nine-point scale (1 = not likely at all; 9 = extremely likely) the probability with which the protagonist was supposed to agree with the 14 items of a questionnaire describing thoughts (including counterfactual thoughts) and feelings related to the emotional experience. The questionnaire (reported in Figure 2) was built in order to capture the protagonist’s possible reactions at the time of evaluating the outcome of his decision in relation to three dimensions:

- self-attribution of the outcome due to free choice (e.g., The situation I am currently in is due to the decision I freely made; If I had chosen differently, my situation would be better now; The responsibility for the situation I am currently in is mine);
- external attribution of the outcome due to forced choice (e.g., The situation I am currently in is due to the decision I was forced to make; At the time of the decision I knew that the outcome of my choice would be worse than I wanted; I know I could not have chosen differently);
- affective evaluation of the current state (e.g., I am happy with the consequences of my choice; I am not happy with the way things turned out).

Four different random sequences including the five InVs, the five emotions, and the 14 items of the questionnaire were built, and the participants were pseudo-randomly assigned to the sequences.

It is worthy to note that the we used two questions on responsibility. The first question asked how responsible the protagonist felt for her own choice when it was made and aimed to evaluate the perceived appraisal of the type of choice; the other question was in the phenomenology questionnaire and asked how responsible she felt for the situation in which she was in at the moment of the evaluation of the decision consequences.

After completing the questionnaire, participants gave back the booklet. They were asked whether they had any questions and then were thanked for their participation.

Before conducting the experiment, a pilot study was carried out with 20 unpaid volunteer employees in order to test the believability of the scenarios, the clarity of the instructions (with particular reference to manipulation check questions), and the perceived importance of the target of the decision-making. No changes were required after this study.

### Results

All data analyses were carried out using SPSS 18.0 IBM software.

#### Manipulation Check

In Table 1, the means and standard deviations of the participants’ responses to the five InVs (importance, responsibility, perception of the freedom of the choice, decision justifiability, and evaluation of present working condition) and the five dependent variables (DVs, regret, disappointment, satisfaction, anger toward oneself, and anger toward circumstances) are shown as a function of the independent variables (IVs, choice, outcome, and time).

**Table 1:** Study 1 – means and standard deviations (S.D.) of intermediate variables and emotions as a function of choice, outcome, and time.

	Free choice				Forced choice				Positive outcome		Negative outcome		Positive outcome		Negative outcome	
	Short term	Long term	Short term	Long term	Short term	Long term	Short term	Long term
**Intermediate variables**								
Decision justifiability	6.67 (2.12)	6.26 (2.00)	6.45 (1.90)	6.26 (1.86)	6.64 (2.11)	6.86 (1.92)	6.67 (2.02)	7.21 (1.84)
Evaluation of present work condition	5.31 (1.68)	6.57 (1.58)	2.29 (1.04)	4.33 (1.98)	3.40 (1.29)	5.26 (1.75)	1.52 (0.59)	3.31 (1.72)
Importance	6.21 (2.18)	7.26 (1.53)	7.14 (2.07)	7.71 (1.18)	6.93 (1.76)	7.00 (1.68)	7.62 (1.34)	7.69 (1.42)
Perception of the freedom of the choice	6.71 (2.51)	7.19 (1.71)	6.98 (2.23)	7.48 (1.57)	3.33 (2.28)	3.26 (2.31)	3.45 (2.58)	3.60 (2.60)
Responsibility	6.86 (2.67)	8.14 (1.46)	7.19 (2.05)	7.98 (1.42)	2.95 (2.43)	3.55 (2.41)	3.40 (2.64)	3.45 (2.80)
**Emotions**								
Anger toward circumstances	4.90 (2.98)	5.19 (2.06)	7.38 (2.11)	6.33 (2.43)	7.45 (1.76)	7.31 (2.09)	7.55 (2.03)	8.57 (0.80)
Anger toward oneself	3.90 (2.54)	5.12 (2.31)	6.95 (2.20)	7.31 (2.01)	4.31 (2.57)	4.07 (2.10)	4.14 (2.39)	5.05 (2.43)
Disappointment	4.69 (2.91)	4.88 (2.24)	7.36 (2.03)	7.33 (1.98)	6.48 (2.19)	6.26 (2.13)	7.05 (2.30)	7.95 (1.64)
Regret	4.79 (2.66)	5.90 (2.23)	7.43 (1.89)	7.64 (2.09)	7.17 (1.78)	6.50 (2.22)	7.00 (2.05)	7.88 (1.67)
Satisfaction	4.81 (2.43)	5.60 (1.80)	2.57 (1.85)	3.00 (2.22)	3.02 (2.33)	4.10 (2.05)	2.12 (1.81)	2.50 (1.69)

The first analysis, a 2 x 2 x 2 MANCOVA, was performed to check whether the manipulated experimental conditions affected the five InVs in the predicted directions. Gender (M = 1; *F* = 0) and age of participants were included as covariates. The multivariate tests were significant for all the three IVs. The univariate tests revealed three main effects due to choice, outcome, and time, and two interaction effects: outcome x choice and time x choice. The main effects were examined through pairwise comparisons with Bonferroni correction; the interaction effects were examined through simple effects analysis with Bonferroni adjustment. Choice affected all the InVs, except importance: responsibility, perception of the freedom of the choice, and positive evaluation of present working condition increased with free choice, while decision justifiability increased with forced choice. Outcome affected only the positive evaluation of present working condition, which increased with positive outcome, and importance, which increased with negative outcome. Time affected the positive evaluation of present working condition, responsibility, and importance, all of which increased in long term. The outcome × choice interaction affected the positive evaluation of present working condition, which increased with positive outcome and free choice compared to positive outcome and forced choice. The time × choice interaction concerned importance, which increased with forced choice compared to free choice in short term, while in long term, there was no difference as a function of the type of choice. No effects were due to gender or age. In Supplementary Table 1, the results of the MANCOVA are reported.

#### Effects of Choice, Outcome, and Time on Emotion Intensity

Subsequently, five 2 x 2 x 2 ANCOVAs were conducted on the five emotions to test the effects of the three IVs on their intensity. Gender and age were included as covariates.

The results (reported in Supplementary Table 2) showed that all emotions were affected by choice and outcome, separately and in interaction; moreover, anger toward oneself and satisfaction were affected also by time, while regret, anger toward oneself, and anger toward circumstances were also influenced by the three-way interaction time x outcome x choice. The main and the interaction effects were examined with the same procedure described for the MANCOVA. In detail, regret, disappointment, and anger toward circumstances increased with forced choice, while anger toward oneself and satisfaction increased with free choice; all emotions increased with negative outcome, except satisfaction, which increased with positive outcome. Anger toward oneself and satisfaction increased in long term. Regret and disappointment increased with forced choice when the outcome was positive, whereas no difference in intensity was found as a function of the type of choice when the outcome was negative. However, by examining these interactions as a function of the type of choice, the results slightly differed for the two emotions: With free choice, the intensity of regret increased when the outcome was negative, while with forced choice, such an intensity was always high irrespective of the outcome. On the contrary, the intensity of disappointment always increased with negative outcome, even if this effect was higher with free than forced choice. In addition, for regret, the two-way interaction was further qualified in light of the three-way interaction: With free choice, its intensity was much higher when the outcome was negative rather than positive, both in short and in long term; instead, with forced choice, there was no intensity difference depending on outcome in short term, whereas in long term, the regret intensity slightly increased when the outcome was negative.

Anger toward oneself increased with free choice compared to forced choice when the outcome was negative, whereas with positive outcome, no difference in intensity was found as a function of the type of choice: The average scores were always quite low. In light of the three-way interaction, this emotion always increased with free choice compared to forced choice, except for short term and positive outcome, where no significant difference emerged as a function of choice: Its intensity was low both in free and forced choice. Anger toward circumstances increased with forced choice in both types of outcome, but this effect was higher with positive outcome. When considering the three-way interaction, this emotion increased with forced choice in all conditions, except for short term and negative outcome, where the intensity did not vary as a function of the type of choice.

Satisfaction increased with free choice but only in positive outcome condition; indeed, no intensity difference was found depending on choice when the outcome was negative.

All emotions except satisfaction increased in females compared to males. No effects were due to age.

#### Phenomenological Dimensions and Moderated Mediation Analyses

To reduce the number of items presented in the questionnaire on the emotional experience, a factor analysis with the principal component extraction method was conducted. Before the analysis, one negative item concerning contentment dimension (“I’m not happy with the way things turned out”) was reverse-coded. Varimax rotation was used after controlling, through Oblimin rotation, that there was no correlation higher than 0.30 among components. Three components with eigenvalue >1, explaining 65.11% of the total variance, were extracted. The three components and the items loading on each of them corresponded to those that had been hypothesized in the construction of the questionnaire. They were labelled *self-attribution*, *external attribution*, and *contentment*. The values of Cronbach’s alpha for each dimension were 0.861, 0.773, and 0.796, respectively. The results are reported in Table 2. The factor scores were saved for the subsequent analyses.

**Table 2:** Study 1 – rotated component matrix of phenomenology questionnaire.

Item	Component			*Self-attribution*	*External attribution*	*Contentment*
1 – I have to blame myself for the choice I made.	0.851	−0.036	−0.202
2 – I have to blame myself for the situation I am currently in.	0.767	0.051	−0.018
3 – The responsibility for the situation I am currently in is mine.	0.764	−0.219	0.262
4 – I am aware that I could have chosen differently.	0.735	−0.253	0.098
5 – The situation I am currently in is due to the decision I freely made.	0.706	−0.357	0.275
6 – If I had chosen differently. my situation would be better now.	0.690	0.219	−0.209
7 – The situation I am currently in is due to the decision I was forced to make.	−0.282	0.729	−0.181
8 – If I could have chosen differently. my current situation would be better.	0.169	0.726	−0.288
9 – At the time of the decision I knew that the outcome of my choice would be worse than I wanted.	0.066	0.705	0.025
10 – I have to blame the external circumstances for the choice I was forced to make.	−0.499	0.609	−0.180
11 – I am aware I could not have chosen differently.	−0.493	0.608	0.055
12 – I am happy with the consequences of my choice.	0.111	−0.070	0.890
13 – Things have gone well.	−0.002	−0.047	0.887
14 – I am not happy with the way things turned out. (reversed item)	−0.061	−0.186	0.699

*The bold values indicate the questionnaire items loading on each component*.

To test the central hypothesis of the study, five moderated mediation analyses were performed. This analysis (Muller et al., 2005; Hayes, 2018a,b; Hayes and Rockwood, 2020) examines whether the putative effect of the independent variable (IV) on the dependent variable (DV) is exerted through intermediate variables – Mediators – and whether the mediated effect differs as a function of the values of other variables included in the design – Moderators – which are supposed to affect the relationship between VI, Mediators, and DV. All moderated mediation analyses were conducted using the PROCESS macro for SPSS 3.1 (Hayes, 2018a), which allows 92 different models of moderation, mediation and moderated mediation analyses to be tested. The macro employs a bootstrapping method for estimating indirect effects, that is, the effects of IV on DV through mediating variables at different values of moderators: 95% bias-corrected confidence intervals were calculated through 10,000 bootstrap samples.

In each analysis, we tested a model (model 10 of PROCESS macro, see Figure 1), according to which the type of choice (IV) should affect, singularly or in interaction with outcome and time (Moderators), the corresponding emotion (DV) through the mediation of 5 variables: responsibility, decision justifiability, and the three dimensions extracted from the factor analysis, that is, self-attribution, external attribution, and contentment. For each analysis, choice (1 = forced choice; 0 = free choice), outcome (1 = negative outcome; 0 = positive outcome), and time (1 = long term; 0 = short term) were included in the model as dummy variables. Gender (1 = male; 0 = female) and age were included as covariates.

All results were examined with reference to the intercept (i.e., free choice, positive outcome, and short term). Concerning the relationship between the IV and/or the moderators and the mediators (which was the same for each analysis), the results were similar to those of the MANCOVA and were reported in Supplementary Material (Supplementary Table 3 and subsequent description).

When mediators were included in the model, the results on the five emotions (reported in Supplementary Table 4) were the following. The probability of regret increased with forced choice, negative outcome, self-attribution, and external attribution, while decreased with contentment. The conditional direct effect of choice on the probability of regret was moderated by outcome and time, being significant only for positive outcome and short time, as the ANCOVA already showed. The conditional indirect effects of choice through self-attribution and external attribution were not moderated by outcome and time, since they were significant for both types of outcome and both in short and long term. More precisely, self-attribution, which decreased with forced choice, increased regret: So, the indirect effect was negative. Instead, external attribution, which increased with forced choice, increased regret: So, the indirect effect was positive. The conditional indirect effect of choice through contentment was moderated by outcome, being significant only with positive outcome, in both values of time. In detail, the probability of contentment, which decreased with forced choice and positive outcome, decreased regret: Thus, the indirect effect was positive.

The probability of anger toward oneself increased with negative outcome, responsibility, and self-attribution and decreased with decision justifiability, contentment, and with the interaction choice x outcome. The negative interaction replicated the results already highlighted by ANCOVA, that is, the effect of negative outcome diminished with forced choice. Choice exerted indirect effects through responsibility and self-attribution and conditional indirect effects through decision justifiability and contentment. In detail, irrespective of the values of outcome or time, forced choice decreased responsibility and self-attribution, which in turn increased anger toward oneself: Thus, the indirect effects were negative and were not moderated by outcome or time. Instead, these variables moderated the indirect effect of forced choice through decision justifiability, which was significant only with negative outcome and long term, and through contentment, which was significant only with positive outcome, both in short and in long term: In these conditions, forced choice decreased contentment, which in turn decreased the probability of anger toward oneself, making the indirect effect positive.

The probability of disappointment increased with negative outcome and external attribution, while decreased with contentment. Choice exerted indirect effects through external attribution and conditional indirect effects through contentment. Specifically, independently on the values of outcome and time, forced choice increased external attribution, which in turn increased the probability of disappointment. Instead, the indirect effect of choice was moderated by outcome, being significant only with positive outcome, in short and long term: In these conditions, forced choice decreased contentment, which in turn decreased the probability of disappointment, making the indirect effect positive.

The probability of anger toward circumstances increased with negative outcome, external attribution, and the choice x time interaction, whereas decreased with self-attribution, contentment, and the choice x outcome interaction. Choice exerted a conditional direct effect through positive outcome and long term: The effect of negative outcome decreased with forced choice, where the probability of the emotion was high in both outcomes (negative interaction choice x outcome); with forced choice, the probability of the emotion increased in the long term (positive interaction choice x time). Choice exerted indirect effects through self-attribution and external attribution and conditional indirect effect through contentment. More precisely, irrespective of the values of outcome and time, forced choice decreased the effect of self-attribution and increased the effect of external attribution. The former dimension decreased anger toward circumstances, whereas the latter increased it. Thus, the indirect effects became positive in both cases. Instead, the indirect effect through contentment was significant only for positive outcome, in both values of time: In these conditions, forced choice diminished contentment, which in turn diminished this emotion, making the conditional indirect effect positive.

The probability of satisfaction decreased with negative outcome and increased with decision justifiability and contentment. Choice exerted only a conditional indirect effect through contentment. Indeed, with positive outcome, in both short and long term, forced choice diminished contentment, which in turn increased satisfaction. Thus, such an effect was negative.

The probability of all emotions, except satisfaction, decreased in males compared to females. No significant effects were due to age.

## Study 2

Study 2 was conducted to investigate regret and other emotional reactions that originate from decision-making resulting in action.

It had the same structure as study 1, except for the scenarios.

### Method

#### Participants

Once again participants were 336 unpaid volunteers, aged between 18 and 60 (Mean = 32.25; S.D. = 11.72). They were recruited in the same way as study 1, and all were workers. Thus, also in this study, participants were equally distributed by gender. More than half of them (55.7%) had a high school diploma, 31.8% had a college degree, and 12.5% had completed compulsory education.

#### Materials and Procedure

The eight scenarios (choice x outcome x time) were built around the following plot: The protagonist chooses to leave a promising basketball career to work in a company. In the free choice conditions, s/he does not feel like facing the risks and uncertainties of a sports career and prefers to opt for a safer job. S/he therefore leaves basketball and finds a job with a solid company. In the forced choice conditions, a physical accident prevents the protagonist from continuing his sports career and he is therefore forced to seek a job that is compatible with his changed physical conditions. Finally, he finds employment with a solid company. In the positive outcome conditions, the protagonist realizes that the new job, although a bit monotonous, leaves him free time to cultivate his interests and is also well paid. In the negative outcome conditions, the protagonist realizes that her job does not highlight her skills, is monotonous, and does not provide for career advancement. In all conditions, the protagonist learns that his place on the basketball team has been taken by another player who has also turned out to be very good. The outcome is evaluated a few days (short-term conditions) or 1 year (long-term conditions) after the choice. For each experimental condition, half of times the protagonist is a woman; the other half it is a man.

The procedure and the other materials were the same as study 1. Only the 5 manipulation check questions were slightly modified to adapt them to the different scenarios. For example, the question on the responsibility was the following: “To what extent does the protagonist feel responsible to take on another job instead of a sports career?” The question on the decision justifiability was the following “To what extent does the protagonist consider the reasons for taking another job instead of a sports career to be valid?”

Also before this experiment, a pilot study was carried out with 20 volunteer employees in order to test the plausibility of the scenarios and the clarity of the instructions. No changes were needed after this study.

### Results

The same statistical analyses of study 1 were performed also for study 2.

#### Manipulation Check

In Table 3, the means and standard deviations of the participants’ responses to the five intermediate variables and the five emotions are shown as a function of the independent variables.

**Table 3:** Study 2 – means and standard deviations (S.D.) of intermediate variables and emotions as a function of choice, outcome, and time.

	Free choice				Forced choice			
	Positive outcome		Negative outcome		Positive outcome		Negative outcome	
	Short term	Long term	Short term	Long term	Short term	Long term	Short term	Long term
**Intermediate variables**								
Decision justifiability	6.86 (1.80)	7.02 (1.20)	6.29 (1.86)	6.81 (1.38)	6.71 (2.12)	6.62 (2.16)	5.86 (2.25)	6.00 (1.96)
Evaluation of present work condition	6.98 (1.24)	6.31 (1.46)	3.21 (1.80)	4.19 (1.89)	6.48 (1.38)	5.71 (1.67)	2.36 (1.34)	2.86 (1.42)
Importance	7.71 (1.53)	7.05 (1.58)	7.69 (1.30)	7.52 (1.53)	7.17 (1.50)	6.86 (2.02)	7.29 (1.94)	7.31 (1.79)
Perception of the freedom of the choice	6.69 (1.54)	5.24 (2.03)	6.83 (1.48)	5.86 (1.95)	2.12 (1.55)	2.31 (1.52)	2.12 (1.31)	2.17 (1.48)
Responsibility	7.52 (1.27)	6.81 (1.94)	7.17 (1.75)	7.36 (1.69)	2.07 (1.47)	2.76 (2.31)	2.14 (1.49)	3.29 (1.85)
**Emotions**								
Anger toward circumstances	4.43 (2.66)	4.40 (2.30)	5.90 (2.35)	6.33 (2.04)	6.81 (2.06)	7.05 (1.90)	8.19 (1.06)	7.62 (1.75)
Anger toward oneself	3.98 (2.32)	4.02 (2.05)	6.86 (2.08)	6.10 (2.27)	2.88 (1.91)	3.45 (1.81)	4.21 (1.97)	4.43 (2.61)
Disappointment	4.05 (2.11)	4.21 (1.68)	7.24 (2.16)	6.62 (2.12)	5.60 (2.71)	6.31 (2.30)	7.40 (1.91)	7.36 (1.99)
Regret	5.02 (2.23)	4.33 (1.86)	6.98 (2.31)	6.67 (1.97)	6.45 (2.04)	6.71 (1.94)	7.17 (1.83)	7.33 (1.82)
Satisfaction	6.24 (1.19)	5.83 (1.51)	3.24 (1.91)	3.71 (1.86)	5.31 (1.85)	4.86 (2.17)	2.52 (1.53)	2.33 (1.28)

The results of the 2x2x2 MANCOVA conducted on the InVs (importance, responsibility, perception of the freedom of the choice, decision justifiability, positive evaluation of present working condition), with age and gender as covariates, showed that multivariate tests were significant for age, for the three IVs, for choice x time and outcome x time interactions. The univariate tests revealed that choice affected all InVs; outcome affected decision justifiability and evaluation of present working condition; time affected the perception of the freedom of the choice; choice x time affected responsibility and perception of the freedom of the choice; outcome x time affected the evaluation of present working condition. Pairwise comparisons and simple effects analysis, both of them with Bonferroni adjustment, were used to investigate main and interaction effects. All InVs increased with free choice; decision justifiability and positive evaluation of present working condition increased with positive outcome; in short term, the perception of the freedom of the choice augmented. The interaction between choice and time revealed that with free choice, the responsibility did not vary as a function of time, whereas with forced choice, responsibility was higher in long than in short term. On the contrary, the perception of the freedom of the choice was independent of time in the forced choice condition, while it was higher in short than in long term with free choice. The outcome x time interaction showed that, with positive outcome, the evaluation of present working condition did not vary depending on time, whereas, with negative outcome, this evaluation was more positive in long than in short time. The evaluation of the protagonist’s present working condition was more positive as the age of the participants increased. No significant effects were due to gender. In Supplementary Table 5, the results of the MANCOVA are reported.

#### Effects of Choice, Outcome, and Time on Emotion Intensity

The five 2x2x2 ANCOVAs conducted on the five emotions (with gender and age as covariates) to test the effects of the three IVs on their intensity showed that all emotions were affected by choice and by outcome; moreover, regret, disappointment, and anger toward oneself were also affected by choice x outcome interaction. More specifically, regret, disappointment, and anger toward circumstances increased with forced choice, whereas anger toward oneself and satisfaction increased with free choice; all emotions increased with negative outcome, except for satisfaction, which increased with positive outcome. The intensity of regret and disappointment increased with forced choice compared to free choice, when outcome was positive, while no significant difference depending on choice was found when the outcome was negative. Anger toward oneself always increased with free choice compared to forced choice but this effect was higher with negative than with positive outcome. Regret diminished when the participants’ age increased, whereas the opposite effect was found on satisfaction. No significant effects were due to gender. In Supplementary Table 6, the results of the ANCOVAs are reported.

#### Phenomenological Dimensions and Moderated Mediation Analyses

The factor analysis performed to reduce the number of items presented in the questionnaire on the emotional experience yielded similar results to study 1.

Three components with eigenvalue >1, explaining 62.54% of the total variance, were extracted following the same procedure of study 1. The items loading on each component were the same of study 1. Once again, the components were labelled *self-attribution*, *external attribution*, and *contentment*. The values of Cronbach’s alpha for each dimension were 0.850, 0.704, and 0.831, respectively. The results are reported in Table 4. The factor scores were saved for the subsequent analyses.

**Table 4:** Study 2 – rotated component matrix of phenomenology questionnaire.

Item	Component			*Self-attribution*	*External attribution*	*Contentment*
1 – I have to blame myself for the choice I made.	0.852	−0.010	−0.105
2 – I have to blame myself for the situation I am currently in.	0.841	−0.074	−0.114
3 – The responsibility for the situation I am currently in is mine.	0.740	−0.337	0.155
4 – If I had chosen differently, my situation would be better now.	0.674	0.205	−0.260
5 – I am aware I could have chosen differently.	0.642	−0.338	0.096
6 – The situation I am currently in is due to the decision I freely made.	0.637	−0.429	0.273
7 – I have to blame the external circumstances for the choice I was forced to make.	−0.243	0.701	−0.072
8 – I know I could not have chosen differently.	−0.327	0.667	0.000
9 – At the time of the decision I knew that the outcome of my choice would be worse than I wanted.	0.097	0.652	−0.076
10 – The situation I am currently in is due to the decision I was forced to make.	−0.359	0.637	−0.119
11 – If I could have chosen differently, my current situation would be better.	0.213	0.556	−0.358
12 – Things have gone well	0.051	−0.038	0.908
13 – I am happy with the consequences of my choice.	0.048	−0.059	0.901
14 – I am not happy with the way things turned out. (reversed item)	−0.154	−0.253	0.728

*The bold values indicate the questionnaire items loading on each component*.

Also in study 2, five moderated mediation analyses were performed to investigate whether the effect of choice on the five emotions was moderated by outcome or time and was mediated by responsibility, decision justifiability, and the three dimensions extracted from the factor analysis, that is, self-attribution, external attribution, and contentment. The model 10 of the Process macro 3.1 for SPSS (Hayes, 2018a) was tested again (see Figure 1) following the same procedure of study 1.

All results were interpreted with reference to the intercept (i.e., free choice, positive outcome, and short term).

The results concerning the relationship between choice and/or the moderators and the mediators (which was the same for each analysis) were similar to those of the MANCOVA and were reported in Supplementary Material (see Supplementary Table 7 and subsequent description).

After including the mediators in the model, the results on the five emotions were the following.

The probability of regret increased with forced choice, negative outcome, self-attribution, and external attribution, while decreased with contentment, choice × outcome interaction, and the participants’ age. The conditional direct effect of choice on the probability of regret was moderated by outcome, being significant only for positive outcome (as the ANCOVA showed). Indeed, the negative interaction between choice and outcome indicated that with forced choice the effect of negative outcome decreased because in these conditions the probability of regret was high in both outcomes (a result already highlighted by the ANCOVA). The conditional indirect effects of choice through self-attribution and external attribution were not moderated by outcome and time, since they were significant for both types of outcome and both in short and long term. The signs of these effects were the same as in study 1. The conditional indirect effect of choice through contentment was moderated by outcome, being significant only with positive outcome, in both values of time. The sign of this effect was positive, as well as in study 1.

The probability of anger toward oneself increased with negative outcome, responsibility, and self-attribution and decreased with decision justifiability, contentment, and with the choice x outcome interaction. The negative interaction replicated the results already showed by ANCOVA, that is, the probability of this emotion increased with negative outcome, when the choice was free, but this effect decreased when the choice was forced because the intensity of anger toward oneself was low in both outcomes. Choice exerted an indirect effect through self-attribution and a conditional indirect effect through contentment. More specifically, regardless of the values of outcome or time, forced choice decreased self-attribution, which in turn increased anger toward oneself: Thus, the indirect effect was negative and was not moderated by outcome or time. Instead, the indirect effect of choice was moderated by outcome, being significant only with positive outcome, both in short and in long term: Here, forced choice decreased contentment, which in turn decreased the probability of anger toward oneself, making the indirect effect positive.

The probability of disappointment increased with negative outcome and external attribution, while decreased with contentment and with choice × outcome interaction. The latter indicated that with negative outcome, the probability of disappointment increased with free choice but that with forced choice this effect decreased because the intensity of disappointment was quite similar in both outcomes. Choice exerted a positive indirect effect through external attribution and a positive conditional indirect effect through contentment, as well as in study 1.

The probability of anger toward circumstances increased with forced choice, negative outcome, external attribution, and decreased with responsibility and contentment. The effect of choice was moderated by outcome, being significant only with positive outcome in both values of time: With positive outcome, the probability of this emotion was higher in forced than in free choice, while with negative outcome, it was similar in both types of choice.

Choice exerted an indirect effect through external attribution and a conditional indirect effect through contentment. More precisely, irrespective of the values of outcome and time, forced choice increased the effect of external attribution, which in turn increased the probability of this emotion: Thus, the indirect effect was positive. Instead, the indirect effect through contentment was significant only for positive outcome in both values of time: here forced choice diminished contentment, which in turn diminished the probability of anger toward circumstances, making the conditional indirect effect positive.

The probability of satisfaction decreased with negative outcome and increased with contentment and the participants’ age. Choice exerted only a conditional indirect effect through contentment. Indeed, with positive outcome, in both short and long term, forced choice diminished contentment, which in turn increased satisfaction. Thus, such an effect was negative.

The effects of age were found only on regret and satisfaction, whereas no significant effects were due to gender. These results are reported in Supplementary Table 8.

## Discussion

The results of these studies largely support our hypothesis. We will first discuss the results that are common to both of them and then the differences between them. In particular, we will discuss the effect of the temporal dimension on the five emotions afterward, since it was somewhat different in the two studies.

Both studies showed that a forced choice is a relevant source of regret. Regardless of whether the decision-making resulted in action or inaction, regret was more intense when elicited by a forced than a free choice. Indeed, while regret was always high when the outcome was negative, irrespective of the type of choice, forced choice-related regret did not diminish with a positive outcome, contrarily to regret elicited by a free choice. Actually, in both studies, this pattern was similar also for disappointment, a finding already found by some authors (Connolly and Butler, 2006; Matarazzo and Abbamonte, 2008), suggesting that for lay people the two emotions are more similar than both decision theorists and many psychologists have argued. Moreover, neither regret nor disappointment was affected by the two appraisal dimensions we took into account, responsibility for the choice and decision justifiability. Once again, this finding disconfirms the prevailing theoretical perspective on regret which considers responsibility as both a necessary requisite for regret and a discriminant criterion for differentiating it from disappointment (see Theoretical background section). Besides, decision justifiability has been considered as a powerful condition capable of reducing the regret intensity (Connolly and Zeelenberg, 2002), at least as to concerns the amount of regret deriving from deciding badly (Pieters and Zeelenberg, 2005). However, in our studies, responsibility and/or decision justifiability did not affect regret but the two types of anger and satisfaction, as we will discuss later.

Note that in both studies, the manipulation of responsibility, and more generally of all intermediate variables, was successful: Responsibility increased with free compared to forced choice, suggesting that participants understood the meaning of the term correctly. As concerns decision justifiability, this variable increased with forced vs. free choice in the first study, whereas the opposite result emerged from the second study: Anyway, in both studies, the average scores were always high or medium-high, implying that participants felt that the reasons underlying the decision of the scenario’s protagonist were well-founded. Thus, the finding that two appraisal dimensions were irrelevant not only for disappointment but also for regret indicates that responsibility is not a necessary requisite for regret and that the DJT (Connolly and Zeelenberg, 2002) does not take into account all the sources of regret. Our results reveal that the importance of the forced choice has been disregarded. However, our studies suggest that the distinction between regret and disappointment, as well as the other emotions taken into account, can be identified in the phenomenological aspects we investigated. More precisely, the moderated mediation analyses showed that, in conformity with our assumptions, each of the five emotions considered, while having some overlapping characteristics, had a specific profile resulting from the way in which for each of them the relationship between antecedents, appraisal, and phenomenological aspects took shape. As mentioned above, to build the phenomenology questionnaire, we have used some items describing the thoughts and feeling linked to a free choice resulting in a bad outcome, that is, self-blame, upward counterfactuals, awareness of the freedom of the choice, and self-accountability. In addition, we have thought of a version of the same items, which was compatible with a forced choice. Finally, we have used some items describing the affective (positive and negative) correlates of the decision consequences. In both studies, the three phenomenological dimensions (self-attribution, external attribution, and contentment) were affected by the type of choice in the expected direction: Self-attribution increased with free choice and with negative outcome, whereas external attribution increased with forced choice but was independent of the outcome. Contentment increased with free choice and positive outcome.

In both studies, the moderated mediation analyses showed that regret was the only emotion that increased with both self-attribution and external attribution, whereas decreased with contentment as well as all other negative emotions. In addition, it was the only emotion on which choice exerted both a direct (in interaction with outcome and/or time) and an indirect (through phenomenology) effect in both studies. In our opinion, this is the most relevant finding of these studies since it supports the hypothesis that regret derives from two types of choice and that the forced choice is at least as important as the free choice. In addition, it suggests that the emotional experience of regret is more complex than it is generally thought in literature.

Disappointment increased with external attribution but was not affected by self-attribution. Consequently, although this emotion has the same relationship as regret to antecedents and the two appraisals, its phenomenology is primarily focused on awareness of forced choice. This result questions the dominant conception of disappointment because it reveals how central the relationship with the choice, not just with the outcome, is in its phenomenology.

Anger toward circumstances was the other emotion with similar characteristics to regret. In both studies, it increased with forced choice, negative outcome, and external attribution. Nevertheless, in the first study, this emotion decreased with self-attribution, instead of increasing like regret; in the second study, it decreased when responsibility increased, whereas responsibility never affects regret.

The negative emotion that most differed from regret was anger toward oneself. In both studies, it was the only emotion that increased with free choice in the presence of a negative outcome. With a positive outcome, in fact, the emotion decreased with both types of choice. Furthermore, it increased with responsibility, self-attribution, and decreased with decision justifiability, while it was not affected by external attribution. These results suggest that, at least for the participants in our studies, anger toward oneself is the emotion that encompasses the features that the mainstream of the economic and psychological literature attribute to regret. We will discuss this issue later.

As to concerns satisfaction – the only positive emotion we took into account because of manipulation of the outcome – the results are congruent with the predictions derived by appraisal theories: In both studies, it increased with positive outcome, free choice, and contentment; in the first study, it also increased with decision justifiability. As expected, neither of the other two dimensions of phenomenology had any effect on it.

Although the results of the two studies were very similar, some differences regarding the temporal dimension and the two covariates emerged: Now, we will briefly discuss them.

In study 2, time did not affect the intensity of emotions, but did affect responsibility, self-attribution, and external attribution in interaction with choice. Summing up, when the choice was forced, responsibility and self-attribution tended to be lower in the short than in the long term, whereas with free choice, they were always very high. The probability of external attribution increased in the long term with free choice, whereas it was always high in both time measures when choice was forced.

In study 1, the effect of time was more relevant, since it concerned responsibility, the same two phenomenological dimensions, and almost all emotions except disappointment. Responsibility, self-attribution, and external attribution increased in the long term, even if the effect on self-attribution was produced in interaction with choice. The effect of time on regret, anger toward oneself, and anger toward circumstances was moderated by the three-way interaction with choice and outcome. Thus, in neither study did we find sufficiently clear and robust results to make a congruent explanatory hypothesis. Although the temporal dimension was more significant when decision-making resulted in inaction rather than in action, our findings do not support the temporal theory of regret (Gilovich and Medvec, 1994, 1995), according to which regret related to an omission should be more intense in the long than in the short term. In our first study, both regret and the two types of anger showed such a tendency only in specific interactions with choice and outcome. Moreover, as to concerns regret, the difference in intensity due to the passage of time occurred between two experimental conditions where the average scores were particularly low, that is, the conditions with free choice and positive outcome, compared in short and long term. Consequently, it seems more appropriate to infer from our results that, regardless whether regret derives from an omission or a commission, its intensity is independent of temporal dimension.

The other differences between our two studies concern the effect of gender and age. In the first study, all emotions except satisfaction increased in females compared to males, whereas no significant effects were due to age. In study 2, gender did not affect results, but regret diminished with age, whereas satisfaction and contentment increased with it.

To the best of our knowledge, only few studies found a gender difference between action and inaction regrets, in the opposite direction to our studies. In the context of romantic (Roese et al., 2006) or sexual (Kennair et al., 2016) relationships, men tend to feel more regrets for initiatives or opportunities which were not taken (inaction); instead, women tend to regret choosing an inappropriate partner or having casual sex (action). In other domains, such as education, achievement, or social relationships, no gender difference seems to have been discovered (e.g., Beike et al., 2009; Davidai and Gilovich, 2018), although Morrison and Roese (2011) found that men reported more action-focused life regrets than women. Moreover, some studies in the career domain have found that women regret not having followed their calling (Stewart and Vandewater, 1999; Wrzesniewski et al., 2006; Newton et al., 2012). Thus, we speculated that in study 1, women identified more than men with the protagonist of the omission scenarios and attributed a greater intensity of regret to him/her than men did.

As regards the effect of age found in study 2, which shows that as a function of age regret decreased whereas satisfaction and contentment increased, to our knowledge, no other studies have found this effect. The studies that focused on the relationship between regret and subjective well-being in the life cycle (Jokisaari, 2003; Wrosch et al., 2005) or on collecting life regret in a US representative sample (Morrison and Roese, 2011) did not find any age difference in the action effect. The suggestion stemming from the research on emotion regulation, according to which old adults are often more capable to regulate negative emotions than the young (Carstensen et al., 2003; Blanchard-Fields et al., 2004), is hardly plausible, since this effect was limited to action-based scenarios and did not extend to omission-based scenarios. Although this finding does not concern the main goals of our research nor was expected, we think that it deserves to be further investigated in future research.

To conclude this discussion, we would like to focus on two points.

The first concerns the question of whether the difference between our results and the majority of those reported in the literature is merely a matter of terminology. As we wrote above, the studies dealing with terms from everyday life, as in our case, always have to deal with the problem that there is no one-to-one correspondence between the terms as they are used by lay people and as they are conceived in the specialist literature (Nisbett and Wilson, 1977). This is particularly true when referring to emotions (Sabini and Silver, 2005) and when comparing studies conducted in different countries and with different languages (e.g., Giorgetta et al., 2012). However, in response to this possible objection, it should be noted that the assumption underlying the translatability of one language into another is that there is at least partial overlap between the meaning of the terms in different languages, in the sense that they designate similar referents.

Perhaps the terms *rimpianto* (or *rammarico*) and regret are not completely superimposable, nor are the English and Italian terms of the other emotions investigated, therefore in our studies, we have considered the appraisal and phenomenological aspects of emotions to overcome this possible issue. The success of the manipulation check suggests that participants found the proposed stories believable and that they interpreted them correctly. Thus, the emotional reaction attributed to the protagonists of the stories seems to be plausible, and this seems to confer ecological validity to the studies.

The use of the scenario method is the final point on which we would like to focus.

As we wrote in The rationale of the studies section, this method has the advantage of allowing an accurate manipulation of the experimental variables, especially if the object of the studies concerns decisions in a self-relevant domain and not trivial everyday decisions. The latter can easily be the object of behavioral experiments, because the manipulation of the variables related to them does not raise the ethical issues that would arise in manipulating self-relevant variables. However, the fact that emotional reactions are attributed to a third person and not felt in the first person could cast doubts on the correspondence between the participants’ real subjective experience and the one attributed to the scenario protagonist. We think there are several ways to respond to such an objection. The first is that perspective taking has been considered as a natural aptitude of living being, rooted in the brain. Notably, simulation theory (Gordon, 1986; Goldman, 1989, 1992; Gallese and Goldman, 1998) assumes that the natural way of attributing mental states to others rests on the automatic ability of humans to imagine what they would feel in the situation the other person is in and to extend their own thoughts and feelings to the other person. Second, several studies on perspective taking reported in The rationale of the studies section showed that people tend to put themselves in the other person’s shoes, regardless of whether the instructions request to do it. Third, the fact that in the scenario method, people do not feel directly investigated can allow them to express more freely what they would really think or feel in a similar situation.

Finally, even if one admits that there is not a total correspondence between the experience in first and in third person, the scope of our studies was to compare the emotional reactions elicited, or supposed to be elicited, by free vs. forced choice. Since these reactions were investigated through the same method, the comparison between them remains valid regardless of the possible difference between emotions experienced in the first or third person.

## Conclusion

Overall, we believe that our results can contribute to a deeper understanding of regret and other post-decision emotions along three directions: First, they suggest that the conception of regret derived from decision theorists is too binding, since it posits as necessary some requirements which are not. Second, they reveal that the antecedents and phenomenology of regret are broader than it is generally believed. Third, they reveal that decision-making (specially when its object is important for our life) does not elicit a reduced number of sharply separated emotions; rather, it produces a complex emotional constellation, where the different emotions, singularly and/or in combination with the others, constitute the affective responses to the different aspects of the decision-making process. The emotions we took into account capture some of these aspects, although they are far from covering them all.

## Limitations and Future Research

In our opinion, the major limitation of our studies is that we did not consider the possible behavioral consequences of emotional experiences. In this way, we have omitted to investigate the motivational function of emotions (Frijda, 1986; Lazarus, 1991; Roseman, 2011) and, consequently, to examine further differences and similarities between the five emotions considered.

If we had asked the participants what the protagonist of the scenario was thinking of doing to deal with the situation she was experiencing, we would have had a more complete picture of the emotional experiences investigated, since the behavioral consequences would also have been included. Future research should investigate these aspects.

## Data Availability Statement

The datasets presented in this study can be found in online repositories. The names of the repository/repositories and accession number(s) can be found at: https://doi.org/10.6084/m9.figshare.14444417.v1.

## Ethics Statement

The studies involving human participants were reviewed and approved by Department of Psychology - University of Campania Luigi Vanvitelli. The participants provided their written informed consent to participate in this study.

## Author Contributions

OM conceptualized the studies, analyzed the data, and wrote the manuscript. CG and BP conducted literature searches, collected the data, and prepared the figures and tables. LA and GN revised the manuscript. All authors contributed to the article and approved the submitted version.

## Funding

The department of Psychology of the University of Campania Luigi Vanvitelli paid the publication fee.

## Conflict of Interest

The authors declare that the research was conducted in the absence of any commercial or financial relationships that could be construed as a potential conflict of interest.

## Publisher’s Note

All claims expressed in this article are solely those of the authors and do not necessarily represent those of their affiliated organizations, or those of the publisher, the editors and the reviewers. Any product that may be evaluated in this article, or claim that may be made by its manufacturer, is not guaranteed or endorsed by the publisher.

## Footnotes

^1^The term phenomenology designates the set of feeling, thinking, intentions, and action tendencies that characterize the experience of emotion (see for example, Frijda, 1987, 2009; Roseman et al., 1994).

## Supplementary Material

The Supplementary Material for this article can be found online at: https://www.frontiersin.org/articles/10.3389/fpsyg.2021. 783248/full#supplementary-material
