# Supplementary material for: Regret and Other Emotions Related to Decision-Making: Antecedents, Appraisals, and Phenomenological Aspects
Source: Front Psychol. 2021 Dec 16;12:783248. doi: 10.3389/fpsyg.2021.783248 (PMC8718115; doi:10.3389/fpsyg.2021.783248)
Supplement: Supplementary file 1 [file Table_1.docx]

**Table 1** – **Study 1 -** Results of MANCOVA performed on Intermediate variables.

| *Multivariate tests:* | *Pillai’s Trace* |  | *F* | *Sig.* | η_p_^2^ |
| --- | --- | --- | --- | --- | --- |
|  | .793 |  | 246.021 | .000 | .793 |
| *Univariate tests:* |  |  |  |  |  |
| *Factor* | *Dependent Variable* | *d.f.* | *F* | *Sig.* | η_p_^2^ |
| Choice | Decision justifiability | 1 | 4.589 | .033 | .014 |
|  | Evaluation of present work condition | 1 | 55.816 | .000 | .146 |
|  | Importance | 1 | 1.075 | .301 | .003 |
|  | Perception of the freedom of the choice | 1 | 219.124 | .000 | .402 |
|  | Responsibility | 1 | 277.541 | .000 | .460 |
| Outcome | Decision justifiability | 1 | .042 | .837 | .000 |
|  | Evaluation of present work condition | 1 | 188.518 | .000 | .366 |
|  | Importance | 1 | 14.240 | .000 | .042 |
|  | Perception of the freedom of the choice | 1 | 1.074 | .301 | .003 |
|  | Responsibility | 1 | .281 | .597 | .001 |
| Time | Decision justifiability | 1 | .041 | .840 | .000 |
|  | Evaluation of present work condition | 1 | 110.217 | .000 | .253 |
|  | Importance | 1 | 5.792 | .017 | .017 |
|  | Perception of the freedom of the choice | 1 | 1.169 | .280 | .004 |
|  | Responsibility | 1 | 7.415 | .007 | .022 |
| Choice x Outcome | Decision justifiability | 1 | .408 | .524 | .001 |
|  | Evaluation of present work condition | 1 | 4.731 | .030 | .014 |
|  | Importance | 1 | .000 | .994 | .000 |
|  | Perception of the freedom of the choice | 1 | .005 | .944 | .000 |
|  | Responsibility | 1 | .049 | .825 | .000 |
| Choice x Time | Decision justifiability | 1 | 2.924 | .088 | .009 |
|  | Evaluation of present work condition | 1 | .273 | .602 | .001 |
|  | Importance | 1 | 4.919 | .027 | .015 |
|  | Perception of the freedom of the choice | 1 | .582 | .446 | .002 |
|  | Responsibility | 1 | 1.846 | .175 | .006 |
|  | Decision justifiability | 1 | .391 | .532 | .001 |
| Outcome x Time | Evaluation of present work condition | 1 | 1.159 | .282 | .004 |
|  | Importance | 1 | .406 | .524 | .001 |
|  | Perception of the freedom of the choice | 1 | .054 | .817 | .000 |
|  | Responsibility | 1 | 1.112 | .293 | .003 |
| Table 1 (Continued) | | | | | |
|  |  |  |  |  |  |
| Choice x Outcome x Time | Decision justifiability | 1 | .018 | .893 | .000 |
|  | Evaluation of present work condition | 1 | 1.746 | .187 | .005 |
|  | Importance | 1 | .551 | .459 | .002 |
|  | Perception of the freedom of the choice | 1 | .011 | .915 | .000 |
|  | Responsibility | 1 | .010 | .918 | .000 |
| *Covariate* |  |  |  |  |  |
| Gender | Decision justifiability | 1 | 2.089 | .149 | .006 |
|  | Evaluation of present work condition | 1 | .854 | .356 | .003 |
|  | Importance | 1 | 2.025 | .156 | .006 |
|  | Perception of the freedom of the choice | 1 | 3.863 | .050 | .012 |
|  | Responsibility | 1 | 2.637 | .105 | .008 |
| Age | Decision justifiability | 1 | 1.235 | .267 | .004 |
|  | Evaluation of present work condition | 1 | .032 | .859 | .000 |
|  | Importance | 1 | 2.564 | .110 | .008 |
|  | Perception of the freedom of the choice | 1 | 1.416 | .235 | .004 |
|  | Responsibility | 1 | .215 | .643 | .001 |
| Error |  | 326 |  |  |  |

| **Dependent variables** | **Factors** | Sum of squares | d.f. | Mean squares | F | p. | η_p_^2^ |
| --- | --- | --- | --- | --- | --- | --- | --- |
| Anger towards circumstances | Choice | 246.053 | 1 | 246.053 | 57.661 | .000 | .150 |
|  | Outcome | 129.202 | 1 | 129.202 | 30.278 | .000 | .085 |
|  | Choice x Outcome | 28.117 | 1 | 28.117 | 6.589 | .011 | .020 |
|  | Choice x Outcome x Time | 36.134 | 1 | 36.134 | 8.468 | .004 | .025 |
|  | Gender | 54.616 | 1 | 54.616 | 12.799 | .000 | .038 |
| Anger towards oneself | Choice | 178.317 | 1 | 178.317 | 33.543 | .000 | .093 |
|  | Outcome | 191.122 | 1 | 191.122 | 35.951 | .000 | .099 |
|  | Time | 26.077 | 1 | 26.077 | 4.905 | .027 | .015 |
|  | Choice x Outcome | 104.380 | 1 | 104.380 | 19.635 | .000 | .057 |
|  | Choice x Outcome x Time | 23.071 | 1 | 23.071 | 4.340 | .038 | .013 |
|  | Gender | 26.761 | 1 | 26.761 | 5.034 | .026 | .015 |
| Disappointment | Choice | 58.332 | 1 | 58.332 | 12.490 | .000 | .037 |
|  | Outcome | 285.189 | 1 | 285.189 | 61.063 | .000 | .158 |
|  | Choice x Outcome | 44.811 | 1 | 44.811 | 9.595 | .002 | .029 |
|  | Gender | 61.466 | 1 | 61.466 | 13.161 | .000 | .039 |
| Regret | Choice | 35.689 | 1 | 35.689 | 8.607 | .004 | .026 |
|  | Outcome | 163.533 | 1 | 163.533 | 39.438 | .000 | .108 |
|  | Choice x Outcome | 54.827 | 1 | 54.827 | 13.222 | .000 | .039 |
|  | Choice x Outcome x Time | 35.020 | 1 | 35.020 | 8.445 | .004 | .025 |
|  | Gender | 71.261 | 1 | 71.261 | 17.185 | .000 | .050 |
| Satisfaction | Choice | 92.712 | 1 | 92.712 | 22.331 | .000 | .064 |
|  | Outcome | 282.274 | 1 | 282.274 | 67.989 | .000 | .173 |
|  | Time | 37.338 | 1 | 37.338 | 8.993 | .003 | .027 |
|  | Choice x Outcome | 29.306 | 1 | 29.306 | 7.059 | .008 | .021 |

**Table 2** – **Study 1 -** Significant results of the five ANCOVAs performed on the intensity of the five emotions.

**Table 3** – **Study 1** - Results of the moderated mediation analysis (Model 10 – see Figure 1) reporting the effects of Choice (IV) and Outcome and Time (moderators) on Contentment, Decision justifiability, External attribution, Responsibility, Self-attribution (mediators).

| **Contentment** | | | | |
| --- | --- | --- | --- | --- |
| ***Model summary*** | ***R-sq*** | ***F*** | ***p*** | |
|  | .280 | 18.202 | .000 | |
| ***Model*** | ***B*** | ***t*** | ***p*** | ***CI*** |
| Constant | .374 | 1.973 | .049 | [.001; .748] |
| Choice | -.637 | -3.928 | .000 | [-.956; -.318] |
| Outcome | -1.226 | -9.282 | .000 | [-1.485; -.966] |
| Time | .189 | 1.430 | .154 | [-.071; .450] |
| Choice x Outcome | .645 | 3.440 | .000 | [.277; 1.014] |
| Choice x Time | .151 | .803 | .423 | [-.220; .523] |
| Gender | .205 | 2.174 | .030 | [.020; .390] |
| Age | .005 | 1.074 | .283 | [-.004; .014] |
| **Decision justifiability** | | | | |
| **Model summary** | ***R-sq*** | ***F*** | ***P*** | |
|  | .030 | 1.464 | .179 | |
| ***Model*** | ***B*** | ***t*** | ***p*** | ***CI*** |
| Constant | 6.384 | 14.683 | .000 | [5.529; 7.239] |
| Choice | -.051 | -.138 | .891 | [-.782; .680] |
| Outcome | -.095 | -.314 | .753 | [-.690; .500] |
| Time | -.330 | -1.086 | .278 | [-.927; .267] |
| Choice x Outcome | .279 | .650 | .516 | [-.565; 1.124] |
| Choice x Time | .747 | 1.727 | .085 | [-.104; 1.597] |
| Gender | -.312 | -1.445 | .149 | [-.736; .113] |
| Age | .011 | 1.125 | .262 | [-.009; .031] |
| **External attribution** | | | | |
| ***Model summary*** | ***R-sq*** | ***F*** | ***P*** | |
|  | .210 | 12.286 | .000 | |
| ***Model*** | ***B*** | ***t*** | ***p*** | ***CI*** |
| Constant | -.627 | -3.149 | .002 | [-1.018; -.235] |
| Table 3 (Continued) | | | | |
| Choice | .742 | 4.363 | .000 | [.407; 1.077] |
| Outcome | -.249 | -1.800 | .073 | [-.521; .024] |
| Time | .477 | 3.435 | .001 | [.204; .750] |
| Choice x Outcome | .121 | .615 | .539 | [-.266; .507] |
| Choice x Time | -.081 | -.409 | .683 | [-.470; .308] |
| Gender | -.158 | -1.600 | .111 | [-.352; .036] |
| Age | .006 | 1.370 | .172 | [-.003; .016] |
| **Responsibility** | | | | |
| **Model summary** | ***R-sq*** | ***F*** | ***P*** | |
|  | .466 | 40.953 | .000 | |
| ***Model*** | ***B*** | ***t*** | ***P*** | ***CI*** |
| Constant | 6.513 | 12.782 | .000 | [5.510; 7.515] |
| Choice | -3.766 | -8.648 | .000 | [-4.623; -2.909] |
| Outcome | .144 | .405 | .686 | [-.554; .841] |
| Time | 1.091 | 3.068 | .002 | [.392; 1.791] |
| Choice x Outcome | .022 | .043 | .966 | [-.968; 1.011] |
| Choice x Time | -.779 | -1.537 | .125 | [-1.776; .218] |
| Gender | .430 | 1.701 | .090 | [-.067; .927] |
| Age | .005 | .394 | .694 | [-.019; .028] |
| **Self-attribution** | | | | |
| ***Model summary*** | ***R-sq*** | ***F*** | ***p*** | |
|  | .379 | 28.552 | .000 | |
| ***Model*** | ***B*** | ***t*** | ***p*** | ***CI*** |
| Constant | -.048 | -.270 | .788 | [-.394; .299] |
| Choice | -.699 | -4.639 | .000 | [-.995; -.402] |
| Outcome | .369 | 3.007 | .003 | [.128; .610] |
| Time | .576 | 4.680 | .000 | [.338; .818] |
| Choice x Outcome | -.455 | -2.613 | .009 | [-.797; -.112] |
| Choice x Time | -.358 | -2.041 | .042 | [-.702; -.013] |
| Gender | -.011 | -.131 | .896 | [-.183; .161] |
| Age | .004 | .956 | .340 | [-.004; .012] |

**Description of the results reported in Table 3**

The probability of responsibility decreased with forced choice, in both values of outcome and time (negative choice effect not conditional on moderators), and increased at long term (positive time effect). The probability of self-attribution decreased with forced choice in both values of outcome and time (negative choice effect not conditional on moderators), and increased with negative outcome (positive outcome effect), and at long term (positive time effect). However, when choice was free, the probability of self-attribution increased more with negative than positive outcome, whereas it was similar in both outcomes with forced choice (negative interaction choice x outcome). Furthermore, with free choice the probability of self-attribution increased more in long than in short term, while it was similar in both values of time, in forced choice condition (negative interaction choice x time). The probability of external attribution increased with forced choice, in both values of outcome and time, and at long term. The probability of contentment decreased with forced choice, and with negative outcome. The effect of choice was moderated by outcome, being significant only with positive outcome. Indeed, with negative outcome this probability was similar in both types of choice, whereas with positive outcome it increased more with free choice than with forced choice (positive interaction choice x outcome). No significant effects were found on decision justifiability. The only significant effect of covariates was found on contentment, whose probability increased in males compared to females.

**Table 4** – **Study 1 -** Significant results of the five moderated mediation analyses (Model 10 – see Figure 1) testing the effects of Choice (IV), Outcome and Time (moderators), Contentment, Decision justifiability, External attribution, Self-attribution, Responsibility (mediators), gender and age (covariates) on the five emotions.

| **Anger towards circumstances** | | | | |
| --- | --- | --- | --- | --- |
| ***Model summary*** | ***R-sq*** | ***F*** | ***P*** | |
|  | .441 | 21.254 | .000 | |
| ***Model*** | ***B*** | ***t*** | ***p*** | ***CI*** |
| Constant | 7.652 | 12.270 | .000 | [6.425; 8.878] |
| Contentment | -.790 | -6.638 | .000 | [-1.025; -.556] |
| External attribution | .845 | 7.043 | .000 | [.609; 1.081] |
| Outcome | 1.155 | 3.594 | .000 | [.523; 1.787] |
| Self-attribution | -.362 | -2.180 | .030 | [-.688; -.035] |
| Choice x Outcome | -.840 | -2.037 | .043 | [-1.651; -.029] |
| Choice x Time | .863 | 2.125 | .034 | [.064; 1.662] |
| Gender | -.517 | -2.534 | .012 | [-.918; -.116] |
| **Anger towards oneself** | | | | |
| ***Model summary*** | ***R-sq*** | ***F*** | ***P*** | |
|  | .493 | 26.177 | .000 | |
| ***Model*** | ***B*** | ***t*** | ***p*** | ***CI*** |
| Constant | 5.309 | 8.200 | .000 | [4.035; 6.582] |
| Contentment | -.823 | -6.656 | .000 | [-1.066; -.580] |
| Decision justifiability | -.130 | -2.430 | .016 | [-.236; -.025] |
| Outcome | 1.275 | 3.821 | .000 | [.618; 1.931] |
| Responsibility | .132 | 2.284 | .023 | [.018; .246] |
| Self-attribution | 1.021 | 5.928 | .000 | [.682; 1.360] |
| Choice x Outcome | -1.257 | -2.937 | .004 | [-2.100; -.415] |
| Gender | -441 | -2.085 | .038 | [-.858; -.025] |
| **Disappointment** | | | | |
| ***Model summary*** | ***R-sq*** | ***F*** | ***P*** | |
|  | .433 | 20.541 | .000 | |
| Table 4 (Continued) | | | | |
|  |  |  |  |  |
| ***Model*** | ***B*** | ***t*** | ***p*** | ***CI*** |
| Constant | 7.233 | 11.291 | .000 | [5.972; 8.493] |
| Contentment | -.961 | -7.858 | .000 | [-1.202; -.721] |
| External attribution | .732 | 5.938 | .000 | [.489; .974] |
| Outcome | 1.501 | 4.547 | .000 | [.852; 2.151] |
| Gender | -.547 | -2.611 | .010 | [-.959; -.135] |
| **Regret** | | | | |
| **Model summary** | ***R-sq*** | ***F*** | ***P*** | |
|  | .399 | 17.833 | .000 | |
| ***Model*** | ***B*** | ***t*** | ***p*** | ***CI*** |
| Constant | 6.796 | 11.031 | .000 | [5.584; 8.008] |
| Choice | .900 | 2.306 | .022 | [.132; 1.667] |
| Contentment | -0.993 | -8.438 | .000 | [-1.224; -.761] |
| External attribution | .334 | 2.821 | .005 | [.101; .568] |
| Outcome | .864 | 2.722 | .007 | [.240; 1.488] |
| Self-attribution | .482 | 2.938 | .004 | [.159; .804] |
| Gender | -.621 | -3.085 | .002 | [-1.018; -.225] |
| **Satisfaction** | | | | |
| ***Model summary*** | ***R-sq*** | ***F*** | ***P*** | |
|  | .454 | 22.414 | .000 | |
| ***Model*** | ***B*** | ***t*** | ***p*** | ***CI*** |
| Constant | 3.067 | 5.135 | .000 | [1.892; 4.243] |
| Contentment | 1.184 | 10.378 | .000 | [.959; 1.408] |
| Decision justifiability | .097 | 1.969 | .050 | [.000; .195] |
| Outcome | -.949 | -3.084 | .002 | [-1.555; -.344] |

**Table 5 – Study 2** –Results of MANCOVA performed on Intermediate variables.

| *Multivariate tests:* | *Pillai’s Trace* |  | *F* | *Sig.* | η_p_^2^ |
| --- | --- | --- | --- | --- | --- |
|  | .798 |  | 254.414 | .000 | .798 |
| *Univariate tests:* |  |  |  |  |  |
| *Factor* | *Dependent Variable* | *d.f.* | *F* | *Sig.* | η_p_^2^ |
|  | Decision justifiability | 1 | 5.340 | .021 | .016 |
| Choice | Evaluation of present work condition | 1 | 19.161 | .000 | .056 |
|  | Importance | 1 | 3.943 | .048 | 0.12 |
|  | Perception of the freedom of the choice | 1 | 485.182 | .000 | .598 |
|  | Responsibility | 1 | 581.948 | .000 | .641 |
|  | Decision justifiability | 1 | 6.882 | .009 | .021 |
| Outcome | Evaluation of present work condition | 1 | 396.257 | .000 | .549 |
|  | Importance | 1 | 2.288 | .131 | .007 |
|  | Perception of the freedom of the choice | 1 | .505 | .478 | .002 |
|  | Responsibility | 1 | 1.162 | .282 | .004 |
|  | Decision justifiability | 1 | .303 | .583 | .001 |
| Time | Evaluation of present work condition | 1 | 2.133 | .145 | .007 |
|  | Importance | 1 | 3.270 | .071 | .010 |
|  | Perception of the freedom of the choice | 1 | 6.143 | .014 | .018 |
|  | Responsibility | 1 | 2.042 | .154 | .006 |
|  | Decision justifiability | 1 | .910 | .341 | .003 |
| Choice x Outcome | Evaluation of present work condition | 1 | 1.826 | .178 | .006 |
|  | Importance | 1 | .012 | .914 | .000 |
|  | Perception of the freedom of the choice | 1 | 1.354 | .245 | .004 |
|  | Responsibility | 1 | .246 | .620 | .001 |
|  | Decision justifiability | 1 | .784 | .376 | .002 |
| Choice x Time | Evaluation of present work condition | 1 | .247 | .620 | .001 |
|  | Importance | 1 | .447 | .504 | .001 |
|  | Perception of the freedom of the choice | 1 | 14.945 | .000 | .044 |
|  | Responsibility | 1 | 9.138 | .003 | .027 |
|  | Decision justifiability | 1 | .631 | .428 | .002 |
| Outcome x Time | Evaluation of present work condition | 1 | 15.805 | .000 | .046 |
|  | Importance | 1 | 1.592 | .208 | .005 |
|  | Perception of the freedom of the choice | 1 | .118 | .732 | .000 |
|  | Responsibility | 1 | 3.327 | .069 | .010 |
|  | Table 5 (Continued) | | |  |  |
|  | Decision justifiability | 1 | .631 | .428 | .002 |
| Choice x Outcome x Time | Evaluation of present work condition | 1 | 15.805 | .000 | .046 |
|  | Importance | 1 | 1.592 | .208 | .005 |
|  | Perception of the freedom of the choice | 1 | .118 | .732 | .000 |
|  | Responsibility | 1 | 3.327 | .069 | .010 |
| *Covariate* |  |  |  |  |  |
|  | Decision justifiability | 1 | 3.130 | .078 | .010 |
| Gender | Evaluation of present work condition | 1 | .148 | .701 | .000 |
|  | Importance | 1 | 1.196 | .275 | .004 |
|  | Perception of the freedom of the choice | 1 | .158 | .691 | .000 |
|  | Responsibility | 1 | .214 | .644 | .001 |
|  | Decision justifiability | 1 | .809 | .369 | .002 |
| Age | Evaluation of present work condition | 1 | 17.269 | .000 | .050 |
|  | Importance | 1 | 1.127 | .289 | .003 |
|  | Perception of the freedom of the choice | 1 | 1.665 | .198 | .005 |
|  | Responsibility | 1 | .298 | .586 | .001 |
| Error |  | 326 |  |  |  |

**Table 6** – **Study 2 -** Significant results of the five ANCOVAs performed on the intensity of the five emotions.

| **Dependent variables** | **Factors** | Sum of squares | d.f. | Mean squares | F | Sig. | η_p_^2^ |
| --- | --- | --- | --- | --- | --- | --- | --- |
| Anger towards circumstances | Choice | 366.820 | 1 | 366.820 | 86.109 | .000 | .209 |
|  | Outcome | 155.763 | 1 | 155.763 | 36.565 | .000 | .101 |
| Anger towards oneself | Choice | 180.162 | 1 | 180.162 | 39.017 | .000 | .107 |
|  | Outcome | 269.096 | 1 | 269.096 | 58.277 | .000 | .152 |
|  | Choice x Outcome | 35.340 | 1 | 35.340 | 7.653 | .006 | .023 |
| Disappointment | Choice | 109.789 | 1 | 109.789 | 24.072 | .000 | .069 |
|  | Outcome | 364.310 | 1 | 364.310 | 79.878 | .000 | .197 |
|  | Choice x Outcome | 37.209 | 1 | 37.209 | 8.158 | .005 | .024 |
| Regret | Choice | 95.094 | 1 | 95.094 | 24.145 | .000 | .069 |
|  | Outcome | 182.039 | 1 | 182.039 | 46.221 | .000 | .124 |
|  | Choice x Outcome | 51.830 | 1 | 51.830 | 13.160 | .000 | .039 |
|  | Age (covariate) | 36.562 | 1 | 36.562 | 9.283 | .003 | .028 |
| Satisfaction | Choice | 69.305 | 1 | 69.305 | 24.934 | .000 | .071 |
|  | Outcome | 593.332 | 1 | 593.332 | 213.467 | .000 | .396 |
|  | Age (covariate) | 30.977 | 1 | 30.977 | 11.145 | .001 | .033 |

**Table 7** – **Study 2 -** Results of the moderated mediation analysis (Model 10 – see Figure 1) reporting the effects of Choice (IV) and Outcome and Time (moderators) on Contentment, Decision justifiability, External attribution, Responsibility, Self-attribution (mediators).

| **Contentment** | | | | |
| --- | --- | --- | --- | --- |
| ***Model summary*** | ***R-sq*** | ***F*** | ***p*** | |
|  | .469 | 41.414 | .000 | |
| ***Model*** | ***B*** | ***t*** | ***p*** | ***CI*** |
| Constant | .333 | 2.009 | .045 | [.007; .659] |
| Choice | -.321 | -2.306 | .022 | [-.595; -.047] |
| Outcome | -1.412 | -12.308 | .000 | [-1.638; -1.186] |
| Time | .040 | .349 | .727 | [-.187; .268] |
| Choice x Outcome | .198 | -1.229 | .220 | [-.119; .515] |
| Choice x Time | -.107 | -.665 | .507 | [-.425; .210] |
| Gender | .157 | 1.946 | .053 | [-.002; .315] |
| Age | .013 | 3.437 | .001 | [.006; .020] |
| **Decision Justifiability** | | | | |
| **Model summary** | ***R-sq*** | ***F*** | ***P*** | |
|  | .053 | 2.626 | .012 | |
| ***Model*** | ***B*** | ***t*** | ***p*** | ***CI*** |
| Constant | 6.845 | 16.288 | .000 | [6.019; 7.672] |
| Choice | -.100 | -.283 | .777 | [-.794; .594] |
| Outcome | -.348 | -1.195 | .233 | [-.920; .225] |
| Time | .306 | 1.043 | .298 | [-.271; .882] |
| Choice x Outcome | -.388 | -.949 | .343 | [-1.192; .416] |
| Choice x Time | -.360 | -.879 | .380 | [-1.165; .445] |
| Gender | .363 | 1.779 | .076 | [-.039; .764] |
| Age | -.008 | -.815 | .416 | [-.026; .011] |
| **External attribution** | | | | |
| ***Model summary*** | ***R-sq*** | ***F*** | ***P*** | |
|  | .219 | 13.138 | .000 | |
| ***Model*** | ***B*** | ***t*** | ***p*** | ***CI*** |
| Constant | -.922 | -4.589 | .000 | [-1.318; -.527] |
| Table 4 (Continued) | | | | |
| Choice | 1.165 | 6.898 | .000 | [-1.318; -.527] |
| Outcome | .053 | .382 | .702 | [-.221; .327] |
| Time | .446 | 3.179 | .002 | [.170; .721] |
| Choice x Outcome | -.150 | -.765 | .445 | [-.534; .235] |
| Choice x Time | -.394 | -2.012 | .045 | [-.779; -.009] |
| Gender | -.038 | -.386 | .700 | [-.230; .154] |
| Age | .008 | 1.686 | .093 | [-.001; .017] |
| **Responsibility** | | | | |
| **Model summary** | ***R-sq*** | ***F*** | ***P*** | |
|  | .647 | 85.862 | .000 | |
| ***Model*** | ***B*** | ***t*** | ***P*** | ***CI*** |
| Constant | 7.442 | 18.805 | .000 | [6.664; 8.221] |
| Choice | -5.340 | -16.064 | .000 | [-5.994; -4.686] |
| Outcome | .104 | .381 | .704 | [-.435; .643] |
| Time | -.279 | -1.013 | .312 | [-.822; .264] |
| Choice x Outcome | .197 | .512 | .609 | [-.561; .954] |
| Choice x Time | 1.169 | 3.035 | .003 | [.411; 1.927] |
| Gender | -.086 | -.449 | .654 | [.411; 1.927] |
| Age | -.003 | -.327 | .744 | [-.020; .015] |
| **Self-attribution** | | | | |
| ***Model summary*** | ***R-sq*** | ***F*** | ***p*** | |
|  | .471 | 41.699 | .000 | |
| ***Model*** | ***B*** | ***t*** | ***p*** | ***CI*** |
| Constant | .273 | 1.647 | .101 | [-.053; .598] |
| Choice | -1.472 | -10.595 | .000 | [-1.746; -1.199] |
| Outcome | .381 | 3.326 | .001 | [.156; .606] |
| Time | -.206 | -1.782 | .076 | [-.433; .021] |
| Choice x Outcome | -.178 | -1.104 | .270 | [-.494; .139] |
| Choice x Time | .586 | 3.635 | .000 | [.269; .903] |
| Gender | .092 | 1.145 | .253 | [-.067; .250] |
| Age | .007 | 1.903 | .058 | [-.000; .014] |

**Description of the results reported in Table 7**

The probability of responsibility decreased with forced choice in both values of outcome and time (negative choice effect); however, with forced choice, this probability decreased less in long than in short term, whereas with free choice there was no significant difference depending on time values (positive interaction choice x time). No significant effects were found on decision justifiability. The probability of self-attribution decreased with forced choice in both values of outcome and time (negative choice effect) and increased with negative outcome, (positive outcome effect); there also was a positive interaction choice x time, whose interpretation was the same of the one found on responsibility. The probability of external attribution increased with forced choice in both values of outcome and time (positive choice effect) and at long term (positive time effect); there also was a negative interaction choice x time meaning that with free choice this probability decreased more in short than in long term whereas with forced choice it did not depend on time. The probability of contentment decreased with forced choice and with negative outcome, but increased with participants’ age. No significant effects were due to gender. These results are reported in table **7** of Supplementary Material.

**Table 8** **- Study 2** - Significant results of the five moderated mediation analyses (Model 10 – see Figure 1) testing the effects of Choice (IV), Outcome and Time (moderators), Contentment, Decision justifiability, External attribution, Self-attribution, Responsibility (mediators), gender and age (covariates) on the five emotions.

| **Anger towards circumstances** | | | | |
| --- | --- | --- | --- | --- |
| ***Model summary*** | ***R-sq*** | ***F*** | ***P*** | |
|  | .410 | 18.627 | .000 | |
| ***Model*** | ***B*** | ***t*** | ***p*** | ***CI*** |
| Constant | 6.997 | 9.677 | .000 | [5.574; 8.419] |
| Choice | 1.393 | 2.555 | .011 | [.321; 2.465] |
| Contentment | -.364 | -2.516 | .012 | [-.648; -.079] |
| External attribution | .751 | 5.532 | .000 | [.484; 1.018] |
| Outcome | 1.092 | 3.028 | .003 | [.383; 1.802] |
| Responsibility | -.126 | -2.020 | .044 | [-.248; -.003] |
| **Anger towards oneself** | | | | |
| ***Model summary*** | ***R-sq*** | ***F*** | ***P*** | |
|  | .408 | 18.534 | .000 | |
| ***Model*** | ***B*** | ***t*** | ***p*** | ***CI*** |
| Constant | 4.055 | 5.508 | .000 | [2.607; 5.504] |
| Contentment | -.531 | -3.610 | .000 | [-.821; -.242] |
| Decision justifiability | -.115 | -1.997 | .047 | [-.229; -.002] |
| Outcome | 1.282 | 3.491 | .000 | [.559; 2.004] |
| Responsibility | .128 | 2.029 | .043 | [.004; .253] |
| Self-attribution | .958 | 5.707 | .000 | [.628; 1.287] |
| Choice * Outcome | -1.073 | -2.525 | .012 | [-1.910; -.237] |
| **Disappointment** | | | | |
| ***Model summary*** | ***R-sq*** | ***F*** | ***P*** | |
|  | .414 | 19.033 | .000 | |
| ***Model*** | ***B*** | ***t*** | ***p*** | ***CI*** |
| Constant | 6.012 | 8.178 | .000 | [4.565; 7.458] |
| Contentment | -.982 | -6.686 | .000 | [-1.271; -.693] |
| External attribution | .624 | 4.525 | .000 | [.353; .896] |
| Table 8 (Continued) | | | | |
| Outcome | 1.251 | 3.411 | .000 | [.530; 1.972] |
| Choice x Outcome | -1.034 | -2.437 | .015 | [-1.869; -.199] |
| **Regret** | | | | |
| **Model summary** | ***R-sq*** | ***F*** | ***P*** | |
|  | .353 | 14.694 | .000 | |
| ***Model*** | ***B*** | ***t*** | ***p*** | ***CI*** |
| Constant | 6.959 | 9.995 | .000 | [5.590; 8.330] |
| Choice | 1.232 | 2.347 | .020 | [.199; 2.264] |
| Contentment | -0,803 | -5,772 | .000 | [-1.077; -.529] |
| External attribution | .312 | 2.385 | .018 | [.055; .569] |
| Outcome | .932 | 2.685 | .008 | [.249; 1.616] |
| Self-attribution | .515 | 3.242 | .001 | [.202; .827] |
| Time | -.698 | -2.354 | .019 | [-1.281; -.115] |
| Age | -.026 | -2.702 | .007 | [-.044; -.007] |
| Choice x Outcome | -1.254 | -3.118 | .002 | [-2.045; -.463] |
| **Satisfaction** | | | | |
| ***Model summary*** | ***R-sq*** | ***F*** | ***P*** | |
|  | .611 | 42.350 | .000 | |
| ***Model*** | ***B*** | ***t*** | ***p*** | ***CI*** |
| Constant | 3.213 | 6.046 | .000 | [2.167; 4.258] |
| Contentment | 1.204 | 11.333 | .000 | [.995; 1.412] |
| External attribution | -.253 | -2.540 | .012 | [-.450; -.057] |
| Outcome | -.947 | -3.572 | .000 | [-1.468; -.425] |
| Age | .017 | 2.357 | .019 | [.003; .031] |
